# RNA sequencing reveals a transcriptomic portrait of human mesenchymal stem cells from bone marrow, adipose tissue, and palatine tonsils

**DOI:** 10.1038/s41598-017-16788-2

**Published:** 2017-12-07

**Authors:** Kyung-Ah Cho, Minhwa Park, Yu-Hee Kim, So-Youn Woo, Kyung-Ha Ryu

**Affiliations:** 1 Department of Microbiology, College of Medicine, Ewha Womans University, Seoul, 07985 Republic of Korea; 2 Department of Pediatrics, College of Medicine, Ewha Womans University, Seoul, 07985 Republic of Korea

## Abstract

Human mesenchymal stem cells (MSCs) are adult multipotent cells that have plasticity and inhabit the stroma of diverse tissues. The potential utility of MSCs has been heavily investigated in the fields of regenerative medicine and cell therapy. However, MSCs represent diverse populations that may depend on the tissue of origin. Thus, the ability to identify specific MSC populations has remained difficult. Using RNA sequencing, we analyzed the whole transcriptomes of bone marrow-derived MSCs (BMs), adipose tissue-derived MSCs (AMs), and tonsil-derived MSCs (TMs). We categorized highly regulated genes from these MSC groups according to functional gene ontology (GO) classification. AMs and TMs showed higher expression of genes encoding proteins that function in protein binding, growth factor, or cytokine activity in extracellular compartments than BMs. Interestingly, TM were highly enriched for genes coding extracellular, protein-binding proteins compared with AMs. Functional Kyoto Encyclopedia of Genes and Genomes (KEGG) pathway analysis also showed differentially enriched signaling pathways between the three MSC groups. Further, we confirmed surface antigens expressed in common and in a tissue-specific manner on BMs, AMs, and TMs by flow cytometry analysis. This study provides comprehensive characteristics of MSCs derived from different tissues to better understand their cellular and molecular biology.

## Introduction

Mesenchymal stem cells (MSCs) reside in the stroma of tissues such as bone marrow, fat, dermis, and umbilical cord. They were initially considered to be effective in tissue repair by replacing diseased or damaged cells with healthy cells^1^. Because of their potential to regenerate damaged tissues and modulate the immune response, MSCs are currently used in many experimental and clinical studies. Since their first isolation from bone marrow by A.J. Friedenstein^2^, MSCs have been successfully isolated and expanded from diverse tissues, including adipose tissue, skin, tooth pulp, and umbilical cord. In fact, evidence has suggested that MSCs may be present in virtually any vascularized tissue throughout the whole body^3^. Recently, palatine tonsil tissue was identified as a source of MSCs^4^, and researchers have demonstrated tonsil-derived MSCs as promising therapeutics to prevent degenerative and inflammatory diseases^5–7^.

According to the convention of the International Society for Cellular Therapy (ISCT), MSCs from various sources are defined as being (1) plastic-adherent under standard cell culture conditions; (2) multipotent, i.e., able to differentiate into osteoblasts, adipocytes, and chondrocytes *in vitro*; and (3) positive for CD73, CD90, and CD105, and negative for CD11b or CD14, CD19 or CD79α, CD34, CD45, and HLA-DR on the cell surface^8^. These combinations of positive and negative CDs have been widely accepted as a method for identifying human MSCs.

However, the characterization of MSC based on cell surface marker phenotype is problematic because of variation in the expression of different CD markers displayed by MSCs derived from different tissues^9,10^. Moreover, to date no exclusive marker has been found to identify MSCs. Therefore, large scale genome-wide molecular phenotyping of MSCs from diverse tissues is to properly identify these cells and understand their biology. RNA sequencing (RNA-seq) using next-generation-sequencing (NGS) platforms has greatly improved the analysis of whole transcriptomes, allowing for the complete annotation and quantification of a large number genes in a single run^11^. This technology allows detection of known and uncharacterized transcripts, and provides information on alternative and novel splicing events. Here, we analyzed and compared the whole transcriptome of MSC populations isolated from human bone marrow (BMs), adipose tissue (AMs), and palatine tonsil tissue (TMs) to make available a detailed transcriptome portrait of these human MSC populations. Further, we characterized cell surface markers on BMs, AMs, and TMs using lyoplates that contained 242 purified monoclonal antibodies and corresponding isotype controls. Our study provides comparative and comprehensive characterization of MSCs from different tissues of origin.

## Materials and Methods

### Ethics statement and human samples

Human tonsils were obtained from tonsillectomies performed in the Department of Otorhinolaryngology, Head and Neck Surgery at Ewha Womans University Mok-Dong Hospital. Written informed consent was obtained from all patients. The protocol was approved by the research ethics committees of Ewha Womans University Mok-Dong Hospital (IRB #ECT11-53-02) and all experiments were performed in accordance with the approved guidelines and regulations.

### Cell culture

MSCs derived from BM (BMs), adipose tissue (AMs), and palatine tonsil tissues (TMs) were grown to passage 7 in 100-mm tissue culture plates. TMs were isolated and cultured as described^4^. Briefly, the tonsillar tissue was chopped and digested in RPMI-1640 (Invitrogen, Waltham, MA, USA) containing 210 U/mL collagenase type I (Invitrogen) and 10 μg/mL DNase (Sigma Aldrich, St. Louis, MO, USA) for 30 min at 37 °C. Digested tissue was subjected to filtration through a wire mesh, and the cells were then washed twice in RPMI-1640/20% normal human serum (NHS; PAA Laboratories GmbH) and once more in RPMI-1640/10% NHS. From among these cells, mononuclear cells were obtained by Ficoll-Paque (GE Healthcare) density gradient centrifugation. Cells were plated at a density of 10^8^ cells in a T-150 culture flask in DMEM (high glucose, Welgene, Daegu, Korea) containing 10% fetal bovine serum (FBS; Invitrogen), 100 μg/mL streptomycin and 100 U/mL penicillin. After 48 h, non-adherent cells were removed from the medium and adherent mononuclear cells were replenished with new culture medium. These cells were expanded with three to five changes of passage, which took about 4 weeks. In this study, cells at passage 7 were used for experiments. AMs were generously provided by RNL Bio (Seoul, Korea), and BMs were purchased from the Severance Hospital Cell Therapy Center (Seoul, Korea). TMs and AMs were cultured in high glucose DMEM (Welgene) containing 10% FBS, 100 μg/mL streptomycin, and 100 U/mL penicillin. BMs were cultured in low glucose DMEM (Welgene) containing 10% FBS, 100 μg/mL streptomycin, and 100 U/mL penicillin.

### RNA extraction, library construction, and sequencing

RNA was extracted from BMs, AMs, and TMs using QIAzol lysis reagent (Qiagen, Hilden, Germany) and subsequently column purified with an RNeasy mini kit (Qiagen). Purified RNA was treated with DNase I (New England Biolabs, Ipswich, MA, USA) to remove genomic DNA. RNA concentration and integrity of each sample were measured using an Agilent 2100 Bioanalyzer (Santa Clara, CA, USA) with an RNA Integrity Number ≥ 8. cDNA libraries were prepared with 1 μg of starting total RNA using the Illumina TruSeq RNA library kit (Illumina Inc., San Diego, CA, USA). The libraries were amplified via 15 cycles of PCR and the amplified library was sequenced using an Illumina HiSeq. 2000 with 100 bp paired end reads per sample (Macrogen, Seoul, Korea).

### Sequence annotation and identification of differentially expressed (DE) genes

FASTQ-formatted sequencing data were de-multiplexed to assign reads to the originating sample. Reads were mapped to the human genome reference (UCSC hg19) using TopHat v2.0.13. The total mapped read numbers for each transcript were determined and normalized to detect fragments per kilobase of exon per million fragments mapped (FPKMs) using Cufflinks. Genes with more than one zero FPKM value out of the analyzed samples were excluded to filter potentially significant gene expressions. For differential expression gene (DEG) analysis, the values of log2 (FPKM + 1) were calculated, and then normalized by quantile. Transcripts with fold-change values larger than 2 with a *P*-value ≤ 0.05 were included in the analysis as DE genes. Hierarchical clustering analysis was performed using complete linkage and Euclidean distance as a measure of similarity to display the DEG expression patterns. All DEG data analysis was conducted using R 3.2.2 (www.r-project.org).

### Gene ontology and enrichment analysis

Functional groups and pathways encompassing the DEGs were identified based on GO and KEGG Pathway analysis using the Database for Annotation, Visualization, and Integrated Discovery (DAVID v.6.8) software. The threshold was set as modified Fisher Exact *P*-value (EASE score) ≤ 0.05.

### Reverse transcription PCR

Reverse transcription PCR was performed on selected genes to validate RNA-seq results. Total RNA from BMs, AMs, and TMs at passage 7 was extracted using Trizol (Invitrogen, MA, USA). cDNA was synthesized using a First-Strand cDNA Synthesis Kit (TOYOBO, Osaka, Japan) according to the manufacturer’s instructions. The amplicons were generated using primers listed in supplementary Table I.

### Cell surface marker screening of BMs, AMs, and TMs

BMs, AMs, and TMs were harvested and analyzed using the BD Lyoplate Human Cell Surface Marker Screening Panel (BD Biosciences, San Jose, CA, USA) containing 242 purified monoclonal antibodies and corresponding isotype controls. The manufacturer’s staining protocol was followed with slight modifications. The cells were plated into round bottom 96-well plates at a density of 5 × 10^5^ cells per well in fluorescence-activated cell sorting (FACS) buffer containing 10 mM EDTA. Samples were incubated in primary antibody in a 100 μL volume for 30 min on ice, followed by FACS buffer wash. Next, cells were incubated with AlexaFluor 647-conjugated secondary antibodies in 100 μL volume for 30 min on ice and washed in FACS buffer twice. Data for each sample were acquired on a FACSCalibur system (BD Biosciences).

### Preparation of conditioned medium

To generate MSC-conditioned media (MSC-CM), BMs, AMs, and TMs (at passages 7–8) were grown in 100-mm tissue culture plates. At 80–90% confluence, the cells were washed twice with phosphate-buffered saline, and the medium was replaced with serum-free DMEM to generate CM. The medium was collected after 48 hr of culture, centrifuged at 1,300 rpm for 5 min, and passed through a 0.2-μm filter. The CM was concentrated to 20-fold of the original concentration by centrifugal filtration (cut-off of 3 K, Amicon Ultra-15, Millipore, Bedford, MA, USA). The concentrated CM were conducted to ELISA or supplementation in osteoclasts (OCLs) differentiation.

### ELISA

To quantify of osteoprotegerin (OPG) secretion from BMs, AMs, and TMs, conditioned medium was collected and the levels of secreted OPG were determined using human OPG ELISA kit in accordance with the manufacturer’s recommended protocols (Cat.No. E-EL-H1341, Elabscience Biotechnology Co., Wuhan, Hubei, China).

### Osteoclastogenesis

RAW 264.7 cells were cultured at a density of 2 × 10^5^ cells/well in 6-well plates in DMEM containing 10% FBS and antibiotics (10,000 U/ml penicillin, 10 mg/ml streptomycin, and 25 μg/ml amphotericin B in 0.85% NaCl solution) in the presence of 50 ng/ml recombinant mouse RANKL (R&D Systems, Minneapolis, MN, USA) and 20 ng/ml recombinant mouse SDF-1α (R&D Systems). SDF-1 α was replenished daily on the first 2 days during the initial stage of differentiation, while RANKL was replenished once every two days for 6 days. Medium was changed every 2 days. Cultures were maintained at 37 °C in a humidified 5% CO_2_ atmosphere.

The CM from each MSCs (BMs-CM, AMs-CM, and TMs-CM) was supplemented with an equal ratio of MSCs and RAW 264.7 based on seeding numbers of RAW 264.7 cells. For example, when 700 µl of TMs-CM was derived from 2 × 10^6^ TMs, 70 µl of CM from the total 700 µl was used to treat 2 × 10^5^ RAW 264.7 cells. The CM was also newly added when replacing differentiation media.

### Tartrate-resistant acid phosphatase (TRAP) staining

RAW 264.7 cells were fixed with 10% glutaraldehyde for 15 min at 37 °C. After washing twice with phosphate-buffered saline (PBS) pre-warmed to 37 °C, cells were treated with TRAP staining solution (0.2 M sodium acetate, 0.2 M acetic acid, 0.3 M sodium tartrate, 10 mg/ml phosphate disodium salt, 0.1% Triton X-100, and 0.3 mg/ml Fast Red Violet LB (F-3381, Sigma Aldrich) in distilled water) for 10 min at 37 °C. TRAP-positive cells appeared dark red. TRAP-positive multinucleated cells containing three or more nuclei were counted as OCLs.

### Proliferation assay

For the 3-(4,5-dimethylthizol-2-yl) 2,5-diphenyl tetrazolium bromide (MTT, Sigma-Aldrich,) assay, cells (BMs, AMs and TMs) were plated onto 96-well microtiter plates at a density of 10^4^/200 µl in fresh medium and were cultured for 120 h. Every 24 h, 20 µl of MTT (5 mg/ml in phosphate-buffered saline) was added to each well, and the plates were returned to the incubator for an additional 4 h. At the end of the incubation period, the supernatants were discarded by a suction and 200 µl dimethyl sulfoxide was added to all the wells, in order to dissolve the dark blue formazan crystals. The plates were subsequently covered with aluminum foil, gently shaken for 15 min, and read at a wavelength of 570 nm.

On the other hands, BMs, AMs and TMs (10^5^ cells, respectively) were plated into 60-mm culture dishes and incubated at 37 °C in a 5% CO_2_ humidified incubator. After 96 h, the cells were harvested and the number of live cells was counted using the hemocytometer with the trypan blue staining method. The initial seeding cell numbers, duration of culture and final cell numbers were recorded. The doubling time of T-MSC was calculated using the following Patterson formula: doubling time (h) = [{(*T* − *T*_0_)(log_2_)}/(log*N* − log*N*_0_)] where *T* is the time (h) and *N* is the cell count.

### Statistical analysis

Data are presented as mean ± standard error of the mean (SEM). The statistical significance was analyzed by two-way ANOVA or Student’s t-test using GraphPad PRISM 7 software (GraphPad Software Inc., San Diego, CA, USA). For all analyses, a P < 0.05 was considered as statistically significant.

## Results

### RNA-seq analysis

**Figure 1:**
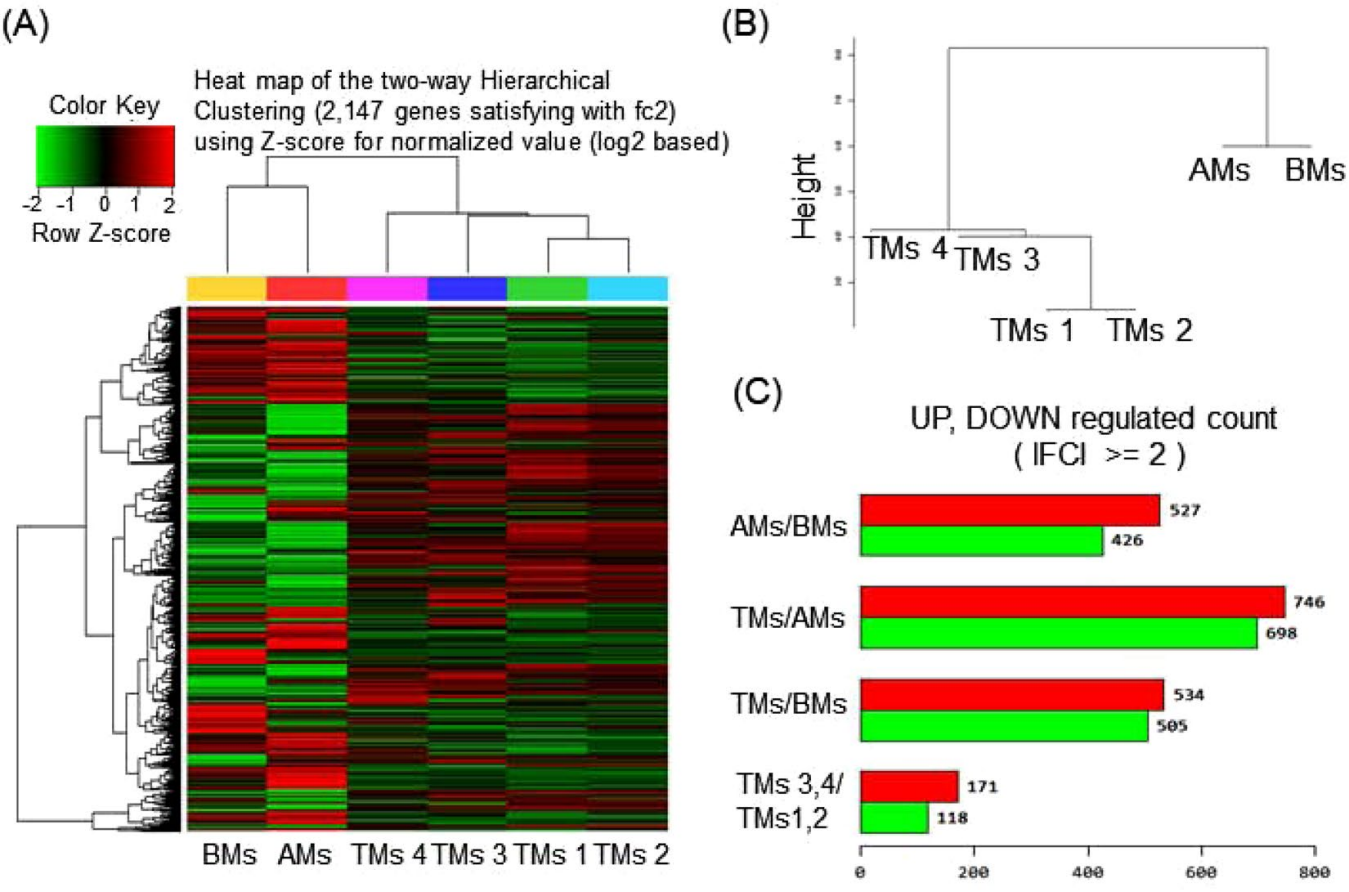
RNA-seq data analysis of BMs, AMs, and TMs. (**A**) Heatmap of hierarchical clustering indicate differentially expressed genes (rows) between BMs, AMs, and four samples of TMs. (fold-change > 2, *P* < 0.05). Red indicates up-regulation and green indicates down-regulation. (**B**) Dendrogram of hierarchical clustering indicates the interclass correlation between the three MSC groups. (**C**) The differentially expressed genes between AMs, BMs, and TMs are shown as up-regulated or down-regulated.

By RNA sequencing, an average of 78 million 100-base long reads from MSCs from each tissue type were mapped to the human reference genome, assembled per gene, and condensed into FPKM expression values, which provided a measure of expression levels for each gene mapped against the human MSC transcriptome. To visualize transcriptomic differences between BM, AM, and TM, a heatmap was generated showing |log_2_ (fold-change)| > 2 (Fig. 1A). To determine whether these gene expression profiles were different between the three MSC groups, we analyzed RNA-seq data of BMs, AMs, and TMs by Pearson correlation coefficient and unsupervised hierarchical clustering (Fig. 1B). The results showed that BMs and AMs were closer in distance to each other than to TMs, and TMs were separated from BMs and AMs. Although four TMs samples showed close distances within samples, we assumed there might be donor variations between TMs samples, as they exhibit paired set and different characteristics. Next, we confirmed the DEG between the three different MSC groups. With the threshold set to “*P* < 0.05, FC > 2”, 953 genes between AMs and BMs, 1444 genes between TMs and BMs, and 1039 genes between TMs and AMs were judged as DEG. Between the four TM samples, 289 genes were differentially expressed between TM 3, 4 and TM 1, 2 (Fig. 1C).

### Functional enrichment analysis of highly regulated genes

**Figure 2:**
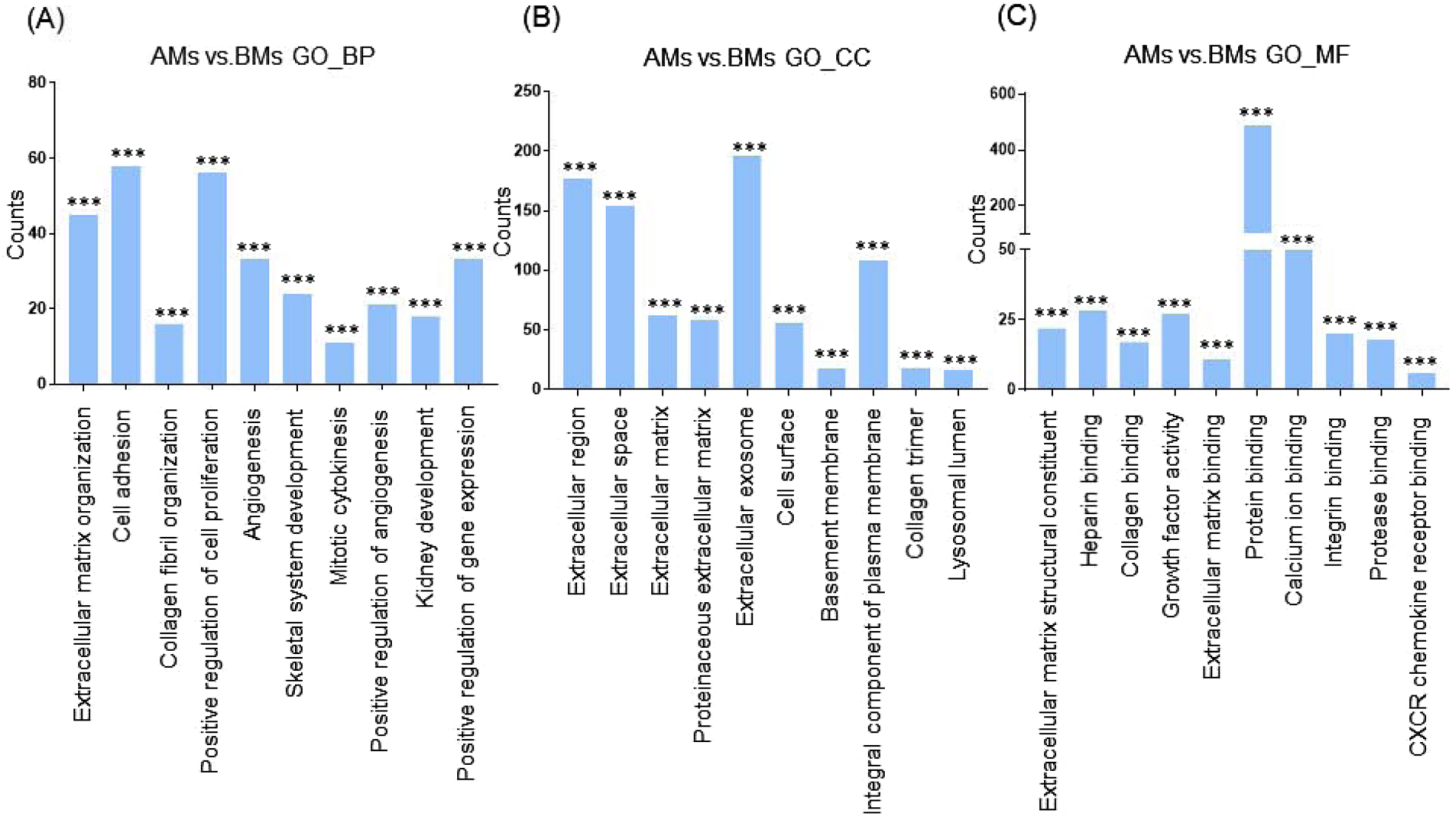
Functional enrichment analysis of highly regulated genes: AMs and BMs. Distribution of gene ontology (GO) terms of DEG between AMs and BMs were annotated in three ontology categories: (**A**) Biological Process, (**B**) Cellular Component, and (**C**) Molecular Function (*P-*value < 0.001).

**Figure 3:**
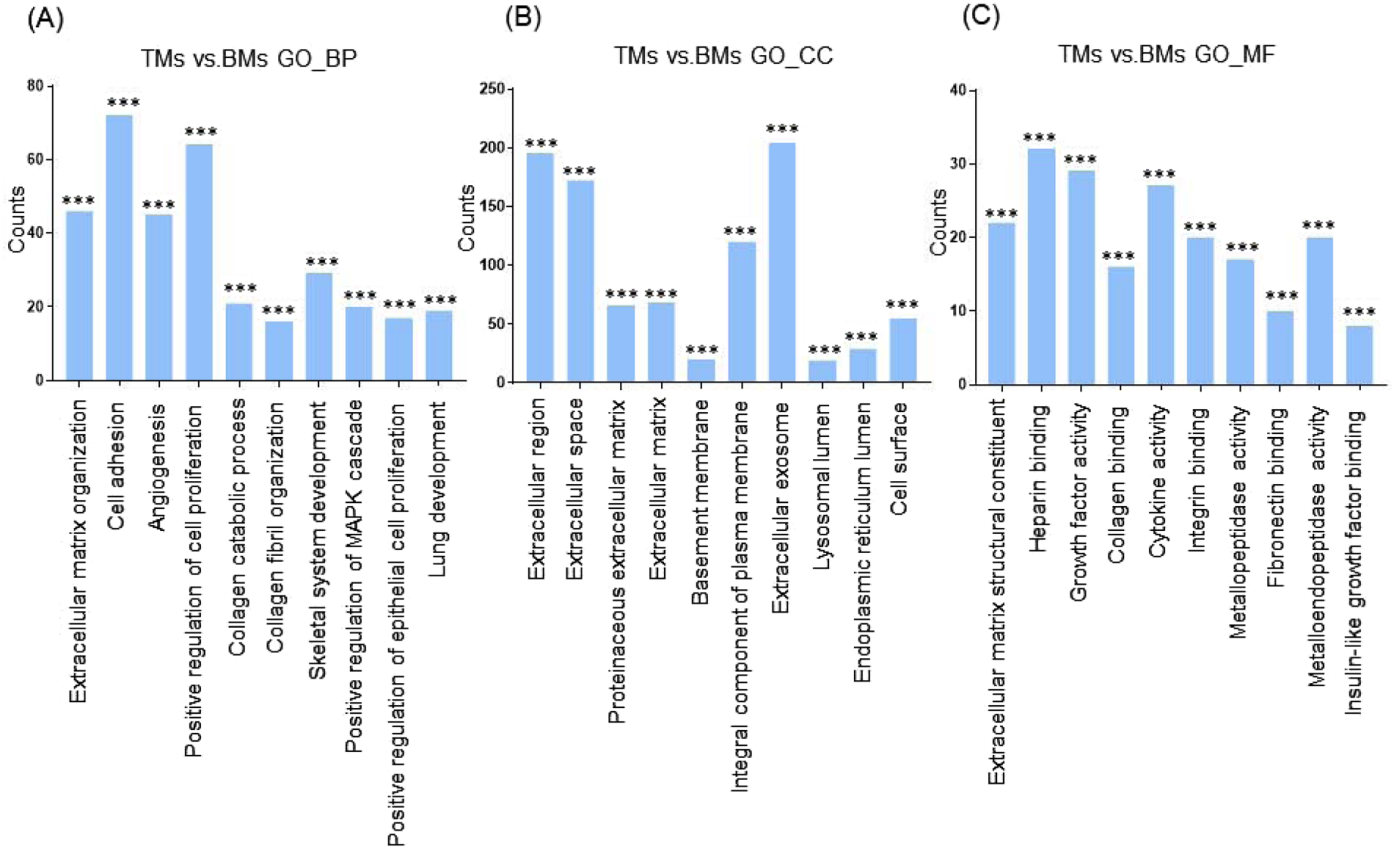
Functional enrichment analysis of highly regulated genes: TMs and BMs. Distribution of gene ontology (GO) terms of DEG between TMs and BMs were annotated in three ontology categories: (**A**) Biological Process, (**B**) Cellular Component, and (**C**) Molecular Function (*P-*value < 0.001).

**Figure 4:**
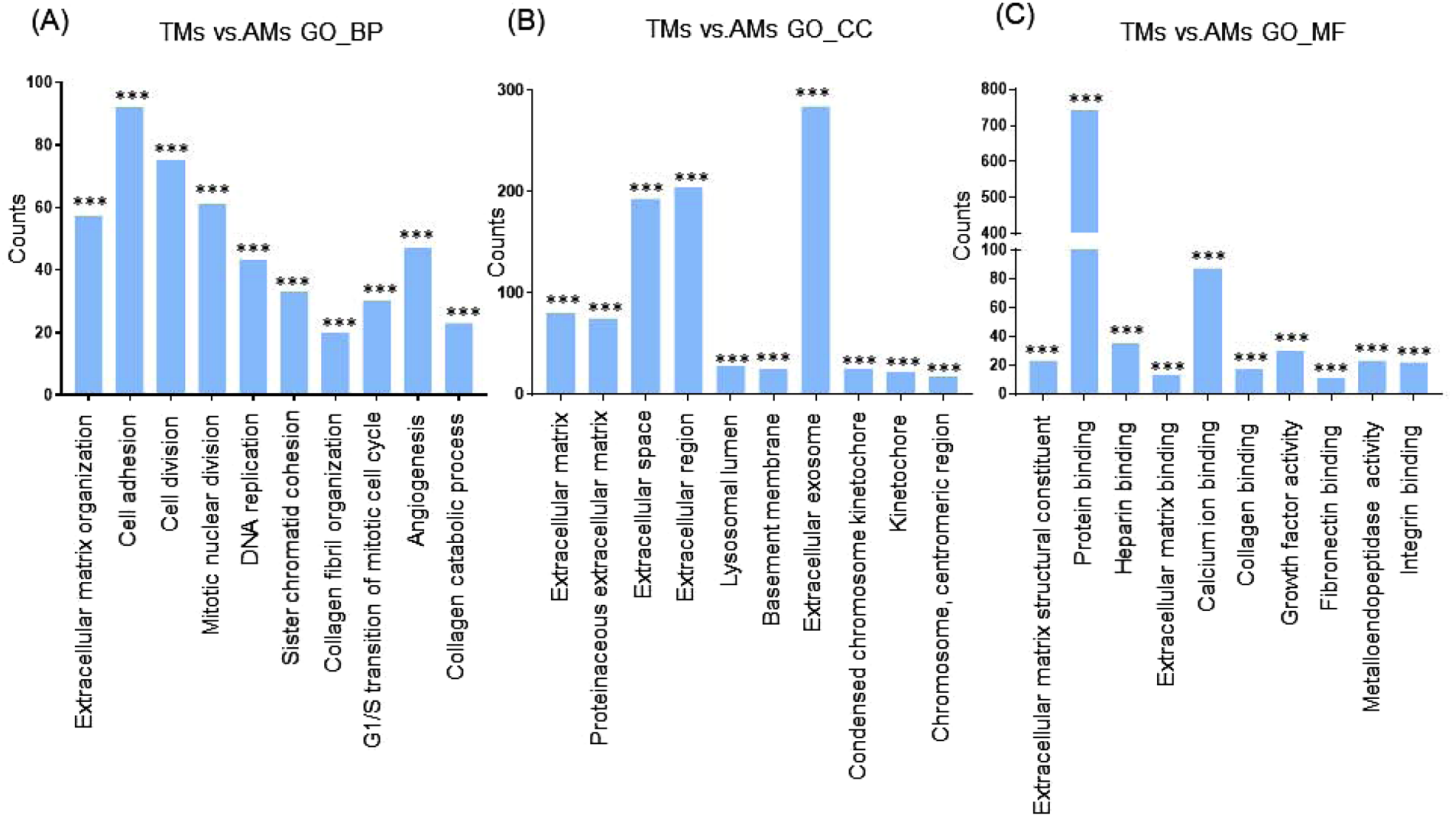
Functional enrichment analysis of highly regulated genes: TMs and AMs. Distribution of gene ontology (GO) terms of DEG between TMs and AMs were annotated in three ontology categories: (**A**) Biological Process, (**B**) Cellular Component, and (**C**) Molecular Function (*P-*value < 0.001).

**Figure 5:**
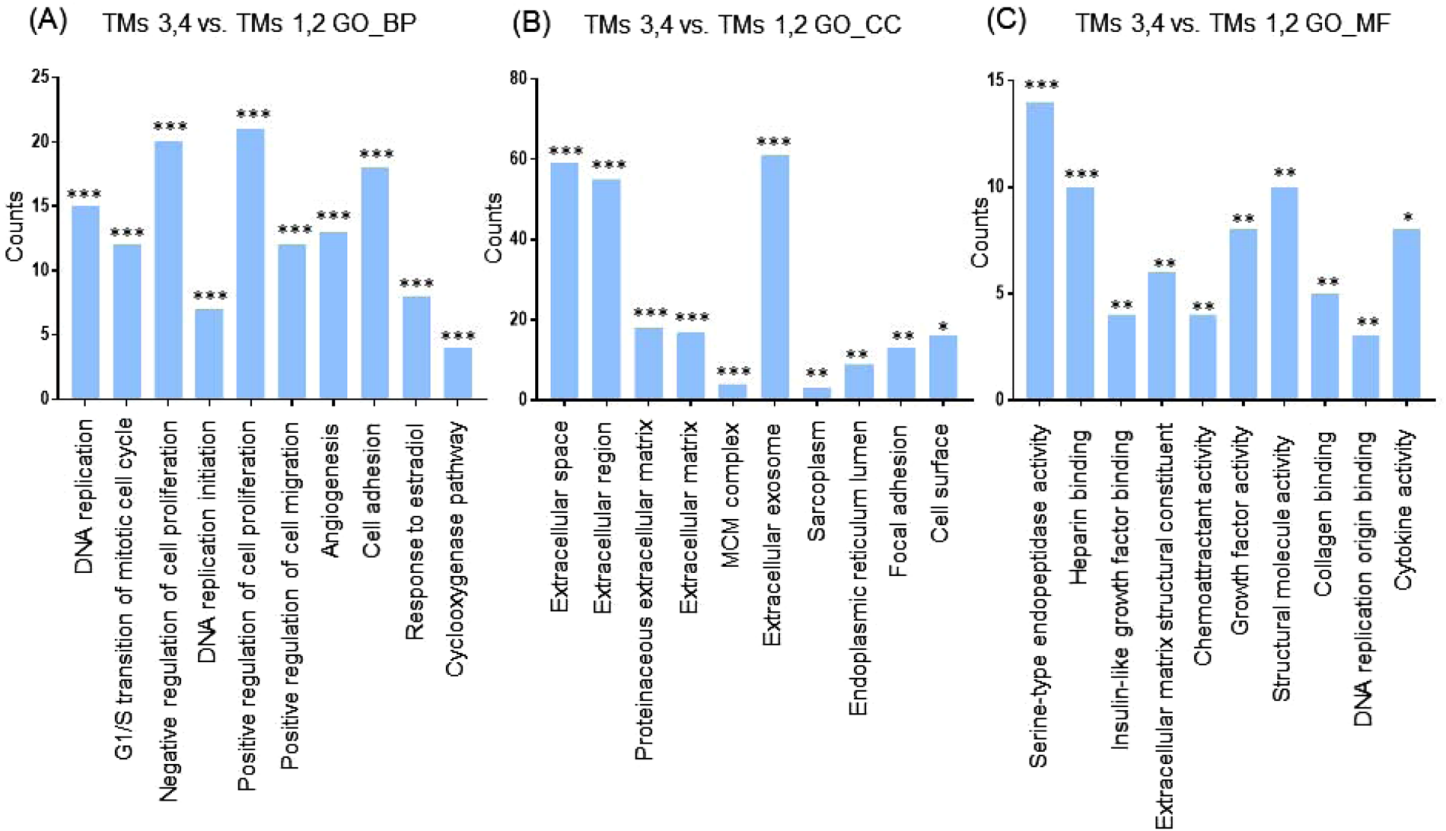
Functional enrichment analysis of highly regulated genes: TMs 3,4 and TMs 1, 2. Distribution of gene ontology (GO) terms of DEG between TMs and AMs were annotated in three ontology categories: (**A**) Biological Process, (**B**) Cellular Component, and (**C**) Molecular Function (*P < 0.05, **P < 0.01, ***P < 0.001).

Next, we categorized the 953 highly differentially regulated genes between AMs and BMs into enriched categories according to GO analysis. The three ontology categories (Biological Process, Cellular Component, and Molecular Function) were annotated and the top 10 categories significantly enriched (*P* < 0.01) within each ontology were described. When we compared the number of genes belonging to each category, the genes in the categories Cell Adhesion, Extracellular Matrix Organization, and Positive Regulation of Cell Proliferation were most highly counted in the “Biological Process” field (Fig. 2A). In the “Cellular Component” section, genes involved in Extracellular Exosome, Extracellular Region, and Extracellular Space were counted as the top three categories (Fig. 2B). In terms of “Molecular Function”, genes associated with Protein Binding, Calcium Ion Binding, and Heparin Binding were most highly distributed (Fig. 2C). When the 1039 genes differentially expressed between TMs and BMs were enriched into categories according to GO analysis, “Biological Process” included the top three categories, Cell Adhesion, Positive Regulation of Cell Proliferation, and Extracellular Matrix Organization (Fig. 3A). In the “Cellular Component”, genes belonging to Extracellular Region, Extracellular Space, and Extracellular Exosome were highly enriched (Fig. 3B). Transcripts coding for proteins associated with heparin binding, growth factor activity, and cytokine activity were enriched as the top three categories in the “Molecular Function” ontology (Fig. 3C). The DEG between TMs and AMs were also clustered. Enriched categories included Cell Adhesion, Cell Division, and Mitotic Nuclear Division in “Biological Process”; Extracellular Exosome, Extracellular Region, and Extracellular Space in “Cellular Component”; and Protein Binding, Calcium Ion Binding, and Heparin Binding in “Molecular Function” (Fig. 4A–C). As we showed hundreds of DEG in TMs, those 289 DEG between TMs 1, 2 and TMs 3, 4 was also analyzed into functional categories. Genes related to regulation of proliferation and cell adhesion showed most high enrichment in the “Biological Process” field (Fig. 5A). In terms of “Cellular Component”, genes involved in Extracellular Exosome, Extracellular Region, and Extracellular Space were counted as the top three categories (Fig. 5B). Serine-type Endopeptidase activity, Heparin Binding, and Structural molecule activity were presented as categories most highly enriched in “Molecular Function” (Fig. 5C).

To evaluate the enrichment in signaling pathways, we performed KEGG analysis. The overlapping signaling pathways in AMs and TMs, which were increased by two folds-changes.

(2FC) over BMs, were the Pertussis, Complement and Coagulation Cascades, and Malaria, Legionellosis, and Rheumatoid Arthritis. In contrast, the Mucin-type O-Glycan Biosynthesis, Pathways in Cancer, PI3K-Akt Signaling Pathway, Focal Adhesion, and ECM-Receptor Interaction were 2FC down-regulated pathways in AMs and TMs compared with BMs (Supplementary Figure 1A and B). The genes in Cell Cycle comprised the most enriched pathway in TMs compared with AMs; this probably represents the proliferative capacity of TMs over AMs that previously reported^6^ (Supplementary Figure 1C). In comparison between TMs, pathways categorized in Staphylococcus aureus Infection, Complement and Coagulation Cascades, and Rheumatoid Arthritis showed high enrichment in 2FC upregulated genes, whereas genes included in Cell Cycle, Pathways in Cancer, Proteoglycans in Cancer pathways were most highly enriched in 2FC downregulation (Supplementary Figure 1D).

The top 50 genes that were up-regulated or down-regulated in TMs compared with BMs or AMs are listed in supplementary Tables II–V. RNA-seq values of selected genes were also verified using RT-PCR (Supplementary Figure 2A). Among validated genes, we noticed TNFRSF11B, a gene encoding OPG. OPG is a protein that inhibits the development of OCLs, acts as a decoy receptor by sequestering RANKL and inhibiting RANK singling^12^. Previously, Kim *et al*. demonstrated that TMs or TMs-CM promote bone mineralization in a mouse model of senile osteoporosis^13^. Because bone loss is accelerated by OCLs, the previous finding that therapeutic potentials of TMs on osteoporosis prompted us to investigate the correlation of TNFRSF11B expression and inhibitory activity of OPG on OCLs differentiation. As shown in Supplementary Figure 2B, the higher expression of TNFRSF11B on TMs were linked to outstanding secretion of OPG protein compared to BMs and AMs. Further, multinucleated OCLs formation was most effectively inhibited in the presence of TMs-CM (Supplementary Figure 2C) Next, we confirmed doubling time and proliferation rate in BMs, AMs, and TMs since TMs expressing genes exhibit higher enrichments in the cluster of cell proliferation and cell division as revealed in the Figs 3 and 4. As shown in the Supplementary Figure 3, TMs showed most shortest doubling time and faster proliferation rate among MSCs.

### Surface marker screening

**Figure 6:**
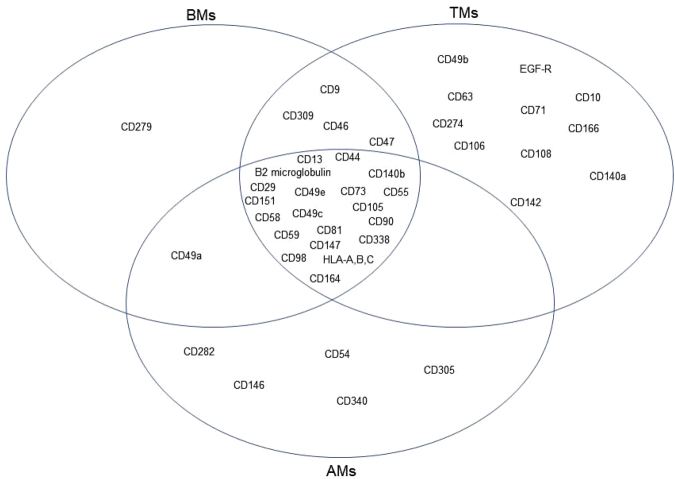
Venn diagrams of surface marker expression on BMs, AMs, and TMs. Lyoplate analysis of surface marker expression on BMs, AMs, and TMs was performed by flow cytometry. The commonly expressed markers among all MSC groups, markers shared between two MSC groups, and tissue-specific markers are shown.

In addition to the transcriptomic characterization of the three MSC groups, we determined the expression profiles of cell surface antigens on BMs, AMs, and TMs (Fig. 6). The cells were harvested at passage 7 and then analyzed by flow cytometry using a surface marker screening panel containing purified monoclonal antibodies specific for 242 human surface proteins. Lyoplate analysis confirmed uniform expression of commonly cited MSC markers CD29, CD44, CD73, CD90, and CD105 in the three MSC groups. BMs, AMs, and TMs also expressed several markers including CD13, β2 microglobulin, CD49C, CD49E, CD55, CD58, C59, CD81, CD98, CD140b, CD147, CD151, CD164, CD338, and HLA-A, -B, and -C. The surface marker expressed only on BMs was CD279. AMs expressed CD54, CD146, CD282, CD305, and CD340, which were not expressed on BMs or TMs. Conversely, CD10, CD49b, CD63, CD71, CD106, CD108, CD142, CD166, CD140a, CD274, and EGF-R were expressed on TMs but not BMs or AMs.

## Discussion

In this study, we used RNA-seq to characterize the genome-wide expression portrait of human MSCs derived from bone marrow (BMs), adipose tissue (AMs), and palatine tonsil (TMs). Further, we determined the phenotypes of BMs, AMs, and TMs to verify common MSC surface markers as well as tissue-specific MSC surface markers by flow cytometric screening of 242 human surface antigens.

MSCs can potentially regenerate mesoderm-derived tissues in adult organs. Their plasticity and immunomodulatory properties have contributed to their widespread use in cell therapy trials as well as to regenerative medicine over the past few years; however, the mechanism of how the molecular machinery defines and channels their behavior still remains poorly understood. Moreover, tissue of origin results certainly factor into the heterogeneity among MSCs. Although BMs have been considered as the gold standard of MSC research, more recently, MSCs from various tissues are being investigated as efficient cellular therapeutics. In particular, recently identified MSCs derived from palatine tonsil (TMs) have revealed excellent differentiation capacity into ectoderm and endoderm components including hepatocytes^14^, islet cells^6^, Schwann cells^15^, and parathyroid cells^16^. This superior differentiation capacity might be due to the tissue of origin. Tonsil, the tissue source for TMs, develops from pharyngeal arch tissue that belongs structurally to the mesothelial and endothelial germ layers. Furthermore, the abundant expression of immunomodulatory proteins and relevant functions of TMs^5,17^ might also come from the tonsil’s function as a secondary lymphoid organ. When considering that different tissue origins might induce the different characteristics of diverse MSCs, the use of genome-wide molecular characterization is indispensable for understanding and determining stem cell identity. In fact, several studies demonstrated that transcriptomic portrait between human MSCs from different origin, for example, bone marrow, placenta, and adipose tissue^18,19^. So far, fibroblasts and MSCs are known to have several similarities including mesodermal differentiation capacity, immunomodulatory functions, and surface makers^20–22^. Nevertheless, those two cell types exploit apparently different molecular mechanisms of differentiation to reach a common cell fates as revealed by RNA-seq analysis^23^. Thus, genomic approach could provide more discriminate characteristics when establishing new source of MSCs.

Our RNA-seq data indicated a closer correlation between BMs and AMs, whereas TMs were more distant from those two MSC groups. This result suggested tissue origin-dependent specificity of MSCs. Another factor, in addition to tissue specificity, that may influence behavior is donor age. In general, bone marrow or adipose tissue is collected in adults, while tonsil is obtained via tonsillectomy, a procedure typically performed on children between 5 and 19 years old. In fact, donor age and gender have been shown to affect function of mesenchymal stem cells in donors even the cells are uniformly derived from BM^24^. Such donor-to-donor variation in the same tissue origin are also shown in our TMs data. Even the variation did not generate independent cluster likely as BMs or AMs, it is necessary to determine causative factors resulting in variation, for example, the inflammatory status at time of tonsillectomy, using TMs from numbers of donors in the future.

The functional enrichment of genes that are highly regulated in AMs or TMs compared with BMs appears to play a significant role in affecting the microenvironment rather than being acted upon, because those genes are highly enriched in categories of proteins that are extracellular, and that bind and communicate with other molecules. When the DEG were categorized into enriched functional signaling pathways, TMs and AMs showed several overlapping pathways of both up-regulated and down-regulated genes compared with BMs, which indicated shared molecular machinery between TMs and AMs. Of note, TMs possess common portrait in comparison with BMs or AMs. Genes included in biological process that higher in TMs are involved in positive cell regulation, cell division, or mitotic nuclear division, as part of the machinery associated to self-renewal. Indeed, we observed shorter doubling time and faster proliferation in culture of TMs compared to AMs or BMs, which is coincided with other previous study^6^. Further, genes highly enriched in the cluster of cell adhesion in TMs rather than BMs or AMs indicate promising source for transplantation into injured tissue.

Thus far, immunophenotypic characterization has been critically used to identify MSCs. Many researchers have demonstrated that surface markers on MSCs vary according to tissue^10^ or even culture status^25^ although some definite markers are already well established. The surface antigen is not only just protein “marking” cells but also is the gate for signaling pathways that affect the proliferative/regenerative capacity of cells. Thus, determining the comprehensive marker profiles of MSCs from different tissues is important for utilizing stem cells with manipulation of specific pathways or homogenously enriched.

BMs, AMs, and TMs uniformly expressed well-known MSCs markers in our lyoplate analysis, but interestingly, AMs or TMs expressed unique surface antigens that were not shared with BMs. These results are consistent with RNA-seq data that showed AMs and TMs possess characteristics distinct from BMs. TMs seem to have diverse roles according to their ability to interact with many other molecules. For example, CD274 is a negative immune modulatory protein that targets immune cells such as T cells^26^. Conversely, TMs also display molecules associated with cell adhesion and migration including CD166 and CD106^27,28^. The migration of MSCs to injured target tissue is an important step in tissue regeneration, thus, the expression of these molecules by TMs may be exploited as therapeutics. Interestingly, one such adhesion molecule, CD146, was expressed only on AMs. In a recent report, adipose tissue-derived MSCs highly express CD146, which is positively related to their growth rate and angiogenic capability^29^. These immunophenotypic features may enable AMs or TMs to participate actively in the microenvironment. However, the lack of such molecules on BMs does not imply lesser “interactive” ability because we did not evaluate inducible expression here. Considering that MSCs have potent plasticity, the intrinsic features should be considered separately from actual performance under various circumstance such as injury or inflammation. Even there was difference in culture condition for three types of MSCs, e.g., glucose concentration, glucose concentration itself might not be a crucial factor to make fundamental differences between MSCs. Because hierarchical clustering showed the close correlation between AMs and BMs even AMs were cultured in high glucose medium and BMs were cultured in low glucose medium. Further, classical makers that should be positive or negative for MSCs identification were turned out as parallel in BMs, AMs, and TMs.

In conclusion, we performed comparative and comprehensive characterization of MSCs from three tissue sources and determined that the transcriptomic and phenotypic profiles differed according to the tissue of origin. In particular, this is the first report to compare genomic portrait of TMs, which is the most recently identified new MSCs, with the BMs and AMs. Although the three types of MSCs share stem cell signatures, TMs exhibit higher enriched genes which could resulting in more proliferative and interactive activity rather than BMs or AMs. The identification of the similarities and differences between MSC populations provide portraits that can be used to accelerate the development of cellular therapeutics based on the specific characteristics of these cells.

## Electronic supplementary material

Supplementary figures and tables

RT PCR full length gel images

## References

[1] Bianco P, Robey PG, Simmons PJ (2008). Mesenchymal stem cells: revisiting history, concepts, and assays. Cell Stem Cell.

[2] Friedenstein AJ, Piatetzky S, Petrakova KV (1966). Osteogenesis in transplants of bone marrow cells. J Embryol Exp Morphol.

[3] Crisan M (2008). A perivascular origin for mesenchymal stem cells in multiple human organs. Cell Stem Cell.

[4] Ryu KH (2012). Tonsil-derived mesenchymal stromal cells: evaluation of biologic, immunologic and genetic factors for successful banking. Cytotherapy.

[5] Kim JY (2017). Tonsil-derived mesenchymal stem cells (T-MSCs) prevent Th17-mediated autoimmune response via regulation of the programmed death-1/programmed death ligand-1 (PD-1/PD-L1) pathway. J Tissue Eng Regen Med.

[6] Kim SY (2015). Characterisation of insulin-producing cells differentiated from tonsil derived mesenchymal stem cells. Differentiation.

[7] Park S (2016). Myogenic differentiation potential of human tonsil-derived mesenchymal stem cells and their potential for use to promote skeletal muscle regeneration. Int J Mol Med.

[8] Dominici M (2006). Minimal criteria for defining multipotent mesenchymal stromal cells. The International Society for Cellular Therapy position statement. Cytotherapy.

[9] Lv FJ, Tuan RS, Cheung KM, Leung VY (2014). Concise review: the surface markers and identity of human mesenchymal stem cells. Stem Cells.

[10] Ong WK (2014). Identification of specific cell-surface markers of adipose-derived stem cells from subcutaneous and visceral fat depots. Stem Cell Reports.

[11] Wang Z, Gerstein M, Snyder M (2009). RNA-Seq: a revolutionary tool for transcriptomics. Nat Rev Genet.

[12] Perez-Sayans M, Somoza-Martin JM, Barros-Angueira F, Rey JM, Garcia-Garcia A (2010). RANK/RANKL/OPG role in distraction osteogenesis. Oral Surg Oral Med Oral Pathol Oral Radiol Endod.

[13] Kim YH (2016). Tonsil-Derived Mesenchymal Stem Cells Promote Bone Mineralization and Reduce Marrow and Visceral Adiposity in a Mouse Model of Senile Osteoporosis. Stem Cells Dev.

[14] Park M (2015). Tonsil-derived mesenchymal stem cells ameliorate CCl4-induced liver fibrosis in mice via autophagy activation. Sci Rep.

[15] Jung, N. *et al*. Tonsil-Derived Mesenchymal Stem Cells Differentiate into a Schwann Cell Phenotype and Promote Peripheral Nerve Regeneration. *Int J Mol Sci* **17**, 10.3390/ijms17111867 (2016). 10.3390/ijms17111867 PMC5133867 27834852

[16] Park YS (2015). Differentiated tonsil-derived mesenchymal stem cells embedded in Matrigel restore parathyroid cell functions in rats with parathyroidectomy. Biomaterials.

[17] Cho KA, Park M, Kim YH, Woo SY, Ryu KH (2017). Conditioned media from human palatine tonsil mesenchymal stem cells regulates the interaction between myotubes and fibroblasts by IL-1Ra activity. J Cell Mol Med.

[18] Jansen BJ (2010). Functional differences between mesenchymal stem cell populations are reflected by their transcriptome. Stem Cells Dev.

[19] Roson-Burgo B, Sanchez-Guijo F, Del Canizo C (2014). & De Las Rivas, J. Transcriptomic portrait of human Mesenchymal Stromal/Stem Cells isolated from bone marrow and placenta. BMC Genomics.

[20] Lorenz K (2008). Multilineage differentiation potential of human dermal skin-derived fibroblasts. Exp Dermatol.

[21] Blasi A (2011). Dermal fibroblasts display similar phenotypic and differentiation capacity to fat-derived mesenchymal stem cells, but differ in anti-inflammatory and angiogenic potential. Vasc Cell.

[22] Haniffa MA (2007). Adult human fibroblasts are potent immunoregulatory cells and functionally equivalent to mesenchymal stem cells. J Immunol.

[23] Jaager K, Islam S, Zajac P, Linnarsson S, Neuman T (2012). RNA-seq analysis reveals different dynamics of differentiation of human dermis- and adipose-derived stromal stem cells. PLoS One.

[24] Siegel G (2013). Phenotype, donor age and gender affect function of human bone marrow-derived mesenchymal stromal cells. BMC Med.

[25] Walmsley GG (2015). High-Throughput Screening of Surface Marker Expression on Undifferentiated and Differentiated Human Adipose-Derived Stromal Cells. Tissue Eng Part A.

[26] Buchbinder EI, Desai A (2016). CTLA-4 and PD-1 Pathways: Similarities, Differences, and Implications of Their Inhibition. Am J Clin Oncol.

[27] Xu L (2016). Cell Adhesion Molecule CD166 Drives Malignant Progression and Osteolytic Disease in Multiple Myeloma. Cancer Res.

[28] Lu ZY (2016). TNF-alpha enhances vascular cell adhesion molecule-1 expression in human bone marrow mesenchymal stem cells via the NF-kappaB, ERK and JNK signaling pathways. Mol Med Rep.

[29] Lee NE (2017). Comparative characterization of mesenchymal stromal cells from multiple abdominal adipose tissues and enrichment of angiogenic ability via CD146 molecule. Cytotherapy.

