# Supplementary material for: RNA sequencing reveals a transcriptomic portrait of human mesenchymal stem cells from bone marrow, adipose tissue, and palatine tonsils
Source: Sci Rep. 2017 Dec 7;7:17114. doi: 10.1038/s41598-017-16788-2 (PMC5719355; doi:10.1038/s41598-017-16788-2)
Supplement: Supplementary file 1 — Supplementary figures and tables [file 41598_2017_16788_MOESM1_ESM.pdf]

RNA sequencing reveals a transcriptomic portrait of human mesenchymal stem cells from bone marrow, adipose tissue, and palatine tonsils.

Kyung-Ah Cho[1], Minhwa Park[1], Yu-Hee Kim[1], So-Youn Woo[1], and Kyung-Ha Ryu[2,*]

[1]Department of Microbiology, College of Medicine, Ewha Womans University, Seoul 07985, Republic of Korea

[2]Department of Pediatrics, College of Medicine, Ewha Womans University, Seoul 07985, Republic of Korea

*Corresponding author:

Kyung-Ha Ryu, MD, PhD

Department of Pediatrics, College of Medicine, Ewha Womans University, Seoul 07985, Republic of Korea

Tel: 82-2-2650-5500

Fax: 82-2-2653-3718

E-mail address: ykh@ewha.ac.kr

Supplementary Figure 1

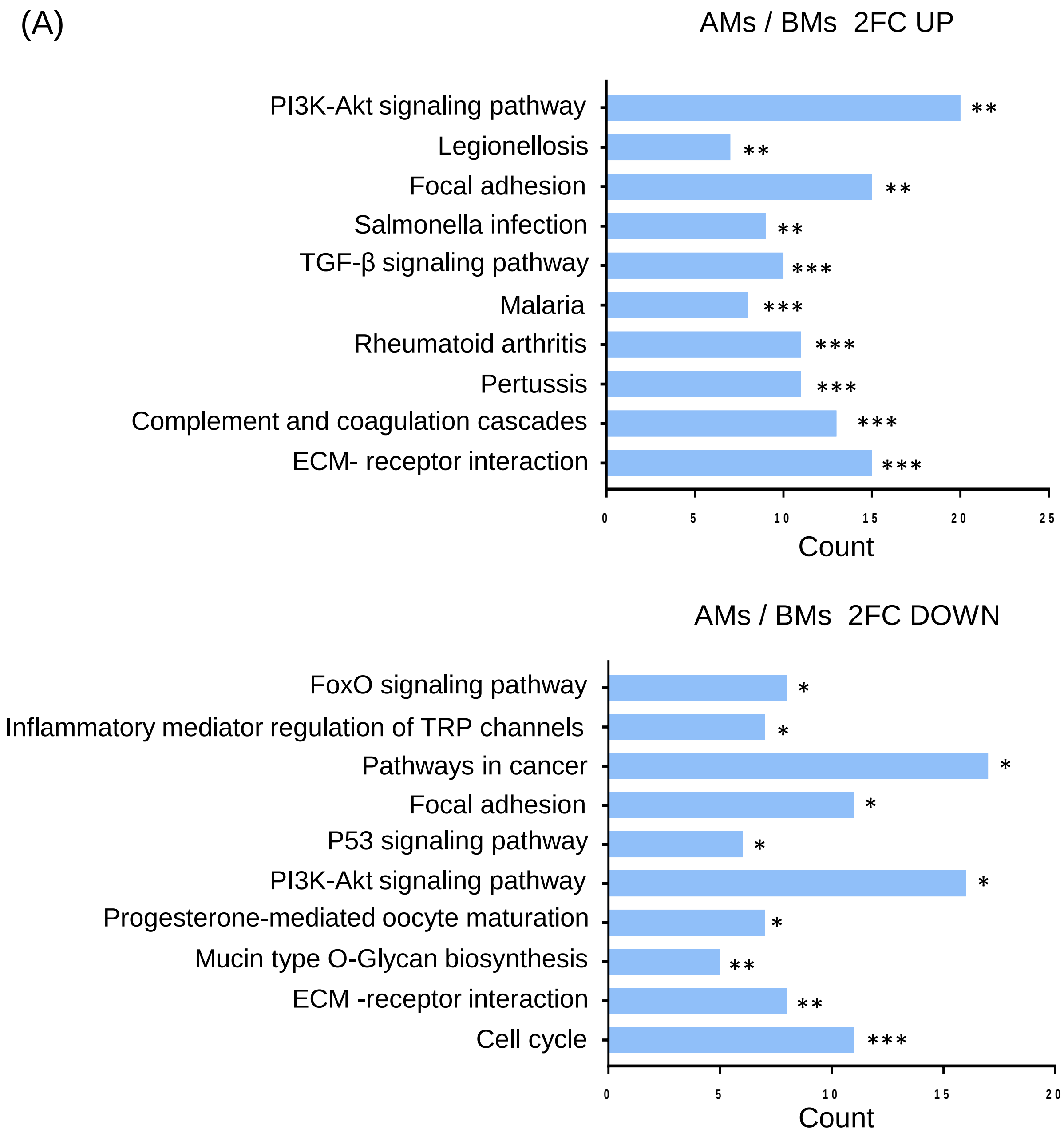

Supplementary Figure 1

(B)

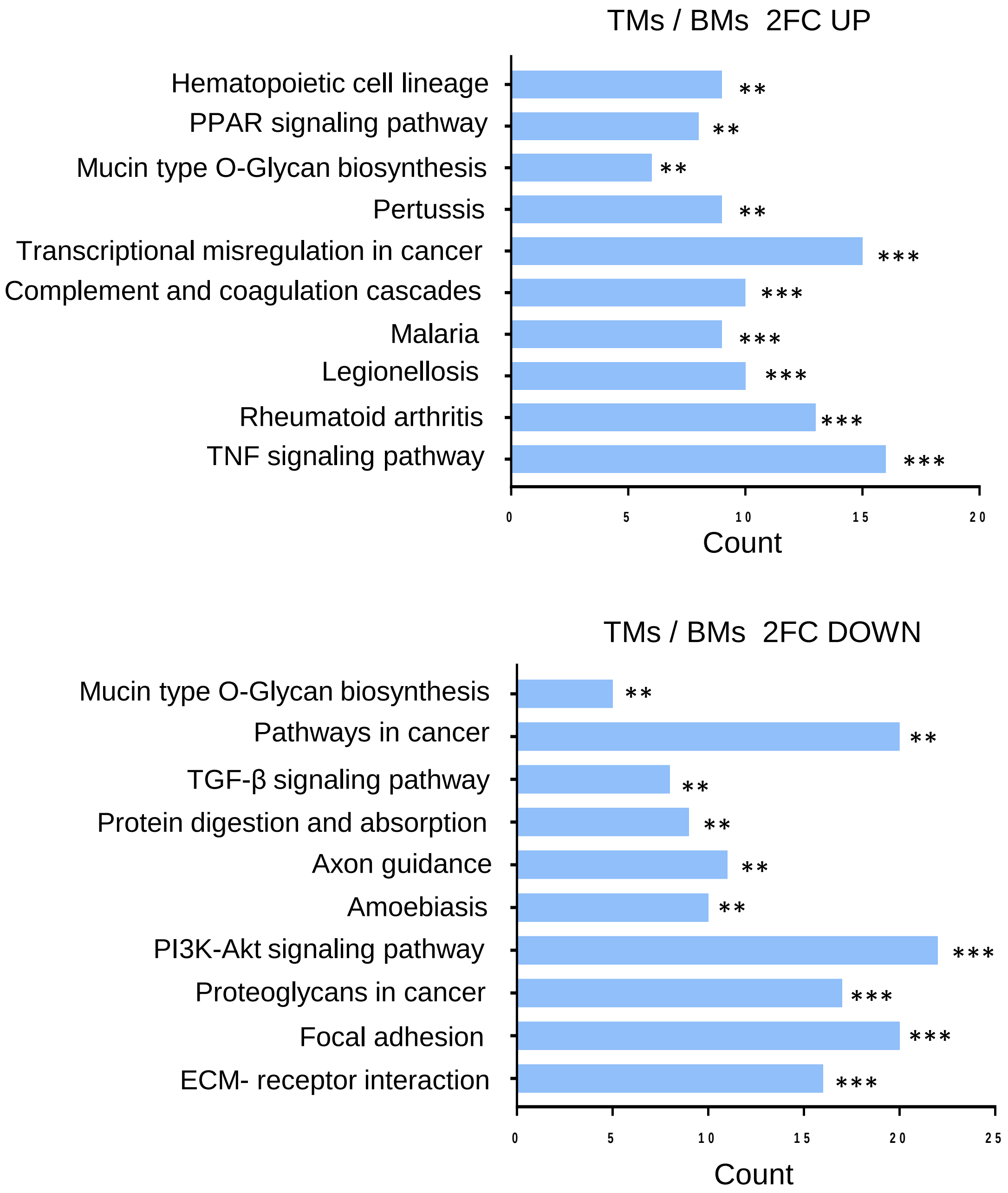

Supplementary Figure 1

(C)

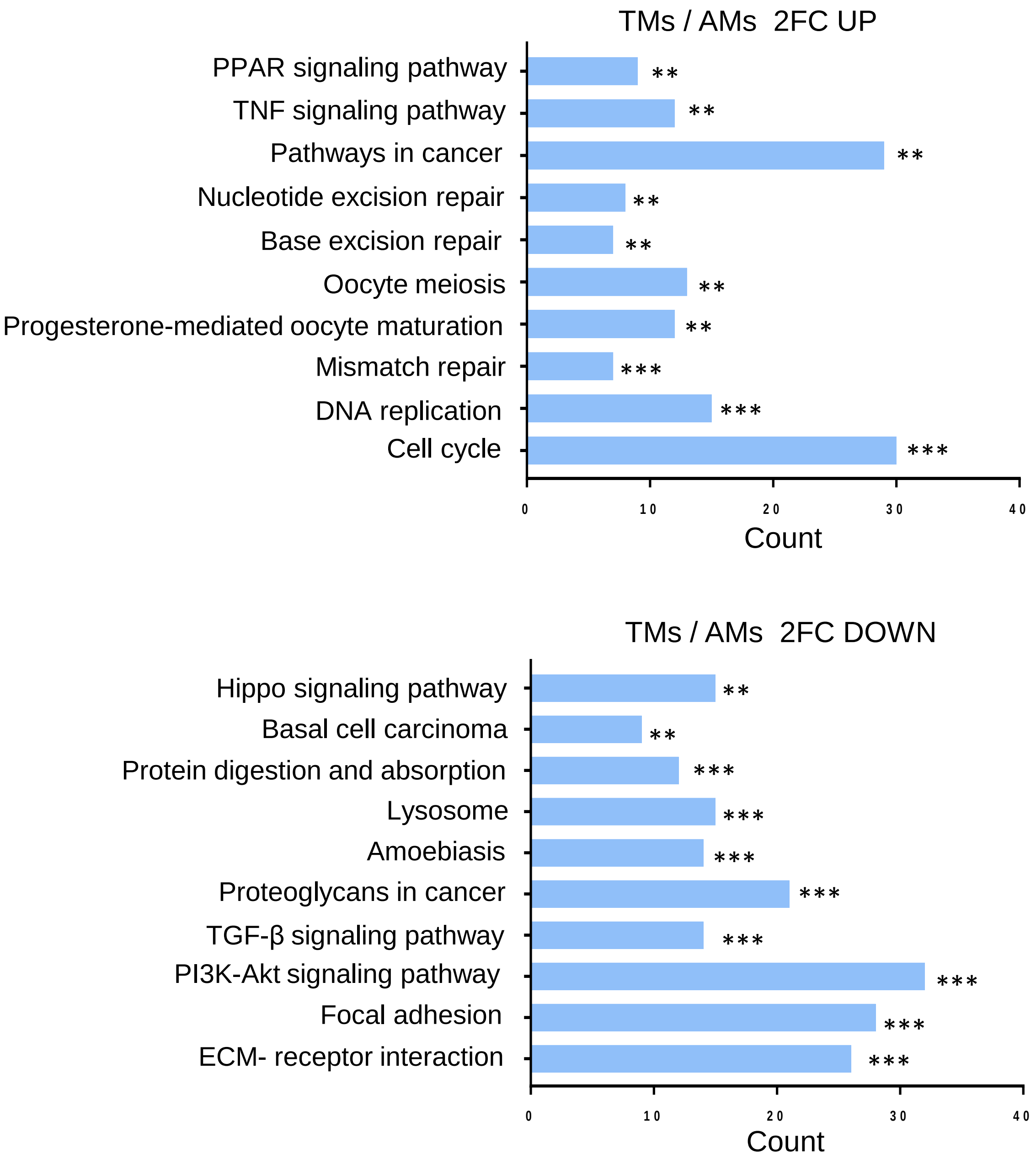

Supplementary Figure 1

(D)

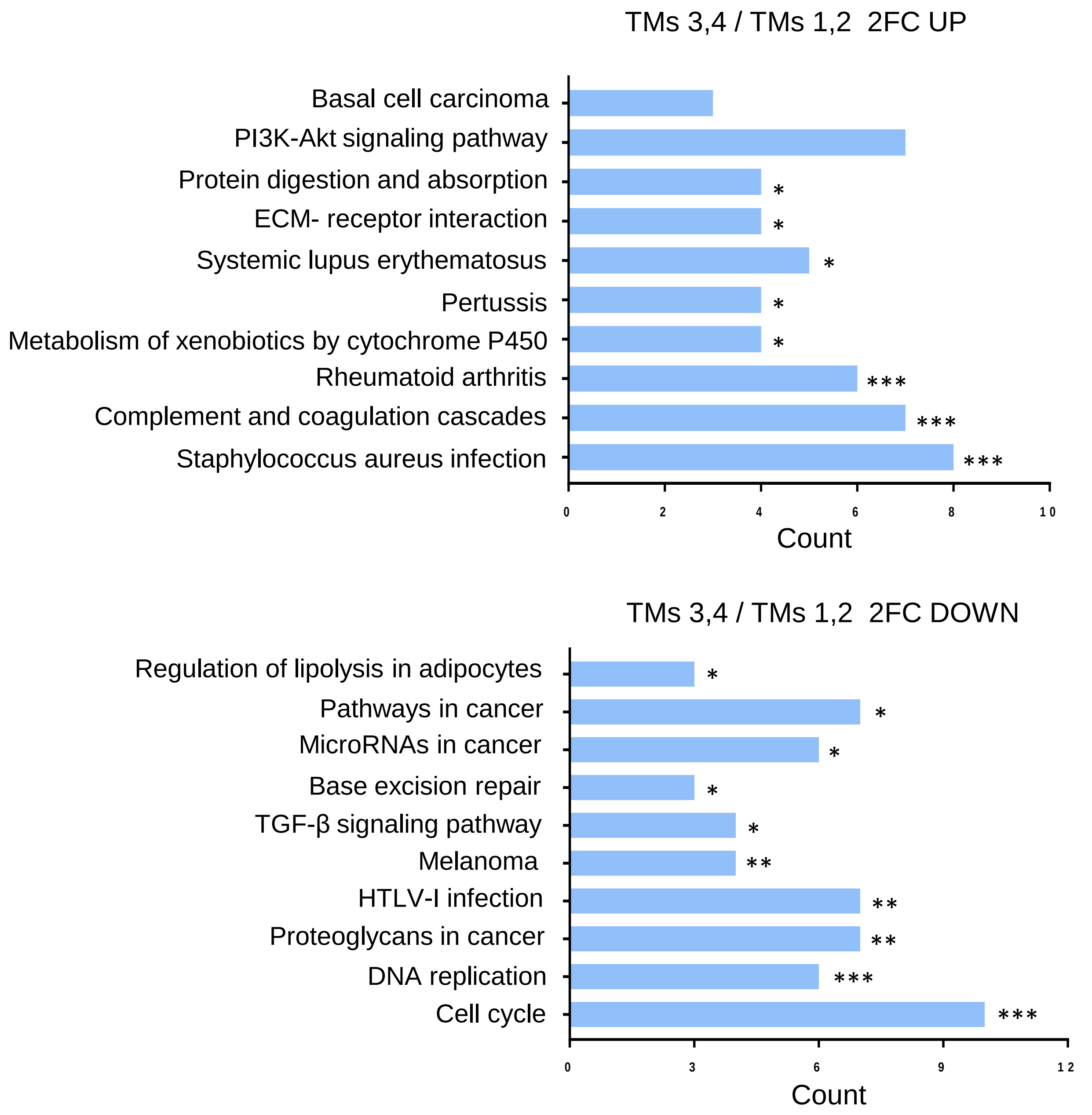

Supplementary Figure 2

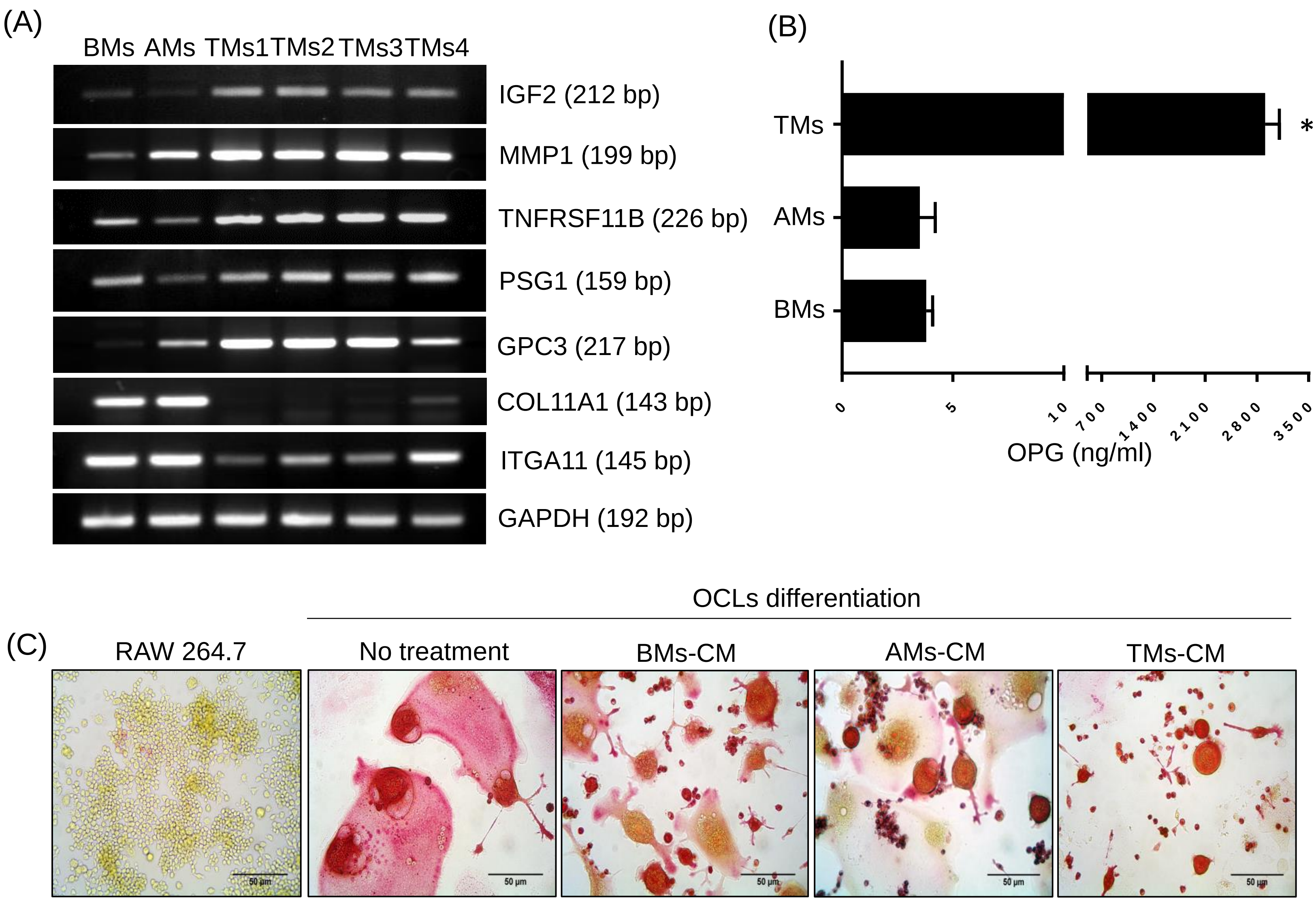

Supplementary Figure 3

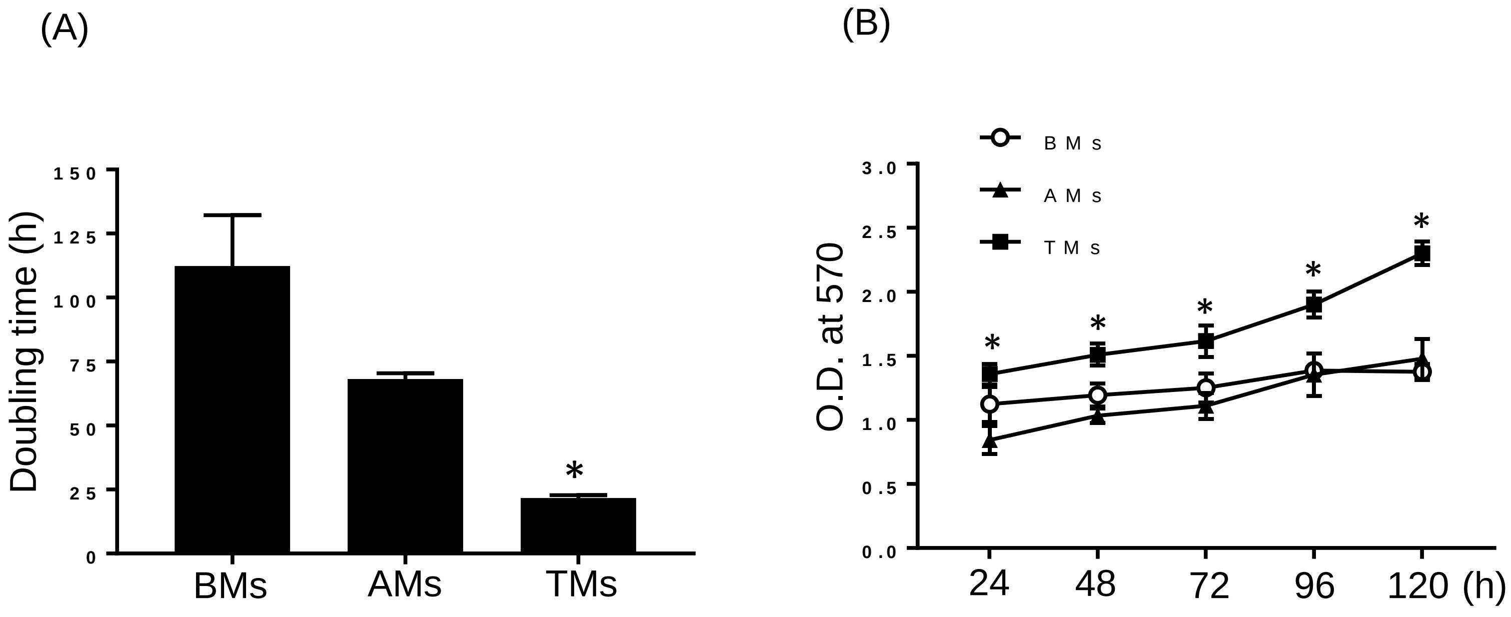

Supplementary Figure Legends

Supplementary Figure 1. Functional KEGG pathway analysis. Up-regulated or down-regulated genes were categorized into enriched functional signaling pathways. (A) The top 10 categories up-regulated (upper) or down-regulated (lower) in AMs compared with BMs are shown (**P* < 0.05, ***P* < 0.01, ***P* < 0.001). (B) The top 10 categories up-regulated (upper) or down-regulated (lower) in TMs compared with BMs are shown (***P* < 0.01, ***P* < 0.001). (C) The top 10 categories up-regulated (upper) or down-regulated (lower) in TMs compared with AMs are shown (***P* < 0.01, ***P* < 0.001). (D) The top 10 categories up-regulated (upper) or down-regulated (lower) in TMs 3,4 compared with TMs 1, 2 are shown (**P* < 0.05, ***P* < 0.01, ****P* < 0.001).

Supplementary Figure 2. Validation of RNA-seq result and functional assay on OCLs differentiation. Upregulated gene particularly in TMs, TNFRSF11B was validated the levels of encoding protein, OPG, and functions. (A) Selected genes were amplified from BMs, AMs, and TMs by RT-PCR to confirm the expression patterns observed in RNA-seq. (B) The protein level of OPG, encoded by TNFRSF11B gene was measured by ELISA in conditioned medium from BMs (BMs-CM), AMs (AMs-CM), and TMs (TMs-CM). The data are presented as the mean ± SEM (**P*<0.05). (C) RAW 264.7 cells were differentiated into OCLs in the presence of BMs-CM, AMs-CM, or TMs-CM and the differentiation efficiency was confirmed by TRAP stain. OCLs were indicated as TRAP-positive multinucleated cells.

Supplementary Figure 3. Comparison of proliferation rate between MSCs. The proliferation rate was analyzed in BMs, AMs, and TMs. (A) The trypan blue method was used to calculate the doubling time of BMs, AMs, and TMs. TMs showed significantly shorter doubling time in comparison with BMs and AMs. The data are presented as the mean ± SEM (**P*<0.05). (B) The proliferation kinetics of BMs, AMs, and TMs were analyzed by MTT assay. 100% was determined based on the result of MTT assay on the seeding day. A significantly

higher proliferation was seen in TMs cultures in comparison with BMs and AMs. The data are presented as the mean ± SEM (*$P<0.05$).

Table I. Primers used for RT-PCR

Primers	Sequences	Product size (bp)
IGF2	F: 5’-ACACCCTCCAGTTCGTCTGT-3’	212
	R: 5’-GGGGTATCTGGGGAAGTTGT-3’	
MMP1	F: 5’-CTCTGGAGTAATGTCACACCTCT-3’	199
	R: 5’- TGTTGGTCCACCTTTCATCTTC-3’	
TNFRSF11B	F: 5’-GCGCTCGTGTTTCTGGACA-3’	226
	R: 5’-AGTATAGACACTCGTCACTGGTG-3’	
PSG1	F: 5’-GACTCCAGACGCAAGCTACC-3’	159
	R: 5’-GCACTCACTGGGTTCCGTAT-3’	
GPC3	F: 5’-CCTTTGAAATTGTTGTTCGCCA-3’	217
	R: 5’-CCTGGGTTCATTAGCTGGGTA-3’	
COL11A1	F: 5’-TAACATCGCTGACGGGAAGTG-3’	143
	R: 5’-CCGTGATTCCATTGGTATCAACA-3’	
ITGA11	F: 5’- GTCACCCTGTCCAACGTGTC-3’	145
	R: 5’-ACATCCCTGTGGTGTAGTAGG-3	
GAPDH	F: 5’-GGTAAAGTGGATATTGTTGCCATCAATG-3’	192
	R: 5’-GGAGGGATCTCGCTCCTGGAAGATGGTG-3’	

Table II. Top 50 transcripts up-regulated in TMs compared to BMs

Transcript_ID	Gene_Symbol	Gene_Description	TM/BM.fc
NM_002421	MMP1	matrix metallopeptidase 1	176.7
NM_002422	MMP3	matrix metallopeptidase 3	136.1
NM_001178096	F3	coagulation factor III	64
NM_001297773	PSG1	pregnancy-specific beta-1-glycoprotein 1	51.6
NM_001271368	PTGS1	prostaglandin-endoperoxide synthase 1	50
NM_001999	FBN2	fibrillin 2	44.5
NM_001511	CXCL1	chemokine (C-X-C motif) ligand 1	40.4
NM_130386	COLEC12	collectin sub-family member 12	28.2
NM_021570	BARX1	BARX homeobox 1	27.2
NM_000584	IL8	interleukin 8	23.1
NM_000599	IGFBP5	insulin-like growth factor binding protein 5	23
NM_015419	MXRA5	matrix-remodelling associated 5	22
NM_004221	IL32	interleukin 32	20.1
NM_001879	MASP1	mannan-binding lectin serine peptidase 1	18.5
NM_198148	CPXM2	carboxypeptidase X (M14 family), member 2	18.5
NM_001193451	TMTC1	transmembrane and tetratricopeptide repeat containing 1	18.2
NM_001198695	MFAP4	microfibrillar-associated protein 4	16.6
NM_003012	SFRP1	secreted frizzled-related protein 1	15.4

NM_001164617	GPC3	glypican 3	15.1
NM_001281534	EPB41L3	erythrocyte membrane protein band 4.1-like 3	14.4
NM_052889	CARD16	caspase recruitment domain family, member 16	13.2
NM_001199640	IL33	interleukin 33	12.9
NM_019554	S100A4	S100 calcium binding protein A4	12.9
NM_000612	IGF2	insulin-like growth factor II	12.9
NM_005382	NEFM	neurofilament	12.7
NM_153202	ADAM33	ADAM metallopeptidase domain 33	11.9
NM_053001	OSR2	odd-skipped related transciption factor 2	11.8
NM_000916	OXTR	oxytocin receptor	11.7
NM_000640	IL13RA2	interleukin 13 receptor, alpha 2	11.3
NM_002546	TNFRSF11B	tumor necrosis factor receptor superfamily, member 11b	11.3
NM_001193335	ASPN	asporin	11.3
NM_001177969	VIT	vitrin	11.2
NM_001124	ADM	adrenomedullin	10.3
NM_000504	F10	coagulation factor X	10.2
NM_000961	PTGIS	prostaglandin I2 (prostacyclin) synthase	10.2
NM_006194	PAX9	paired box 9	10
NM_000902	MME	membrane metallo-endopeptidase	9.7
NM_030965	ST6GALNAC5	ST6 (alpha-N-acetyl-neuraminyl-2,3-beta-galactosyl 1, 3)-N-acetylgalactosaminide alpha- 2,6-sialyltransferase 5	9.1

NM_014059	RGCC	regulator of cell cycle	9.1
NM_016235	GPRC5B	G protein-coupled receptor, family C, group 5, member B	9
NM_001025201	CHN1	chimerin 1	9
NM_018584	CAMK2N1	calcium/calmodulin-dependent protein kinase II inhibitor 1	8.8
NM_002729	HHEX	hematopoietically expressed homeobox	8.4
NM_001992	F2R	coagulation factor II (thrombin) receptor	8.2
NM_000597	IGFBP2	insulin-like growth factor binding protein 2	8.1
NM_001270468	SOCS2	suppressor of cytokine signaling 2	8
NM_001165035	FBLN2	fibulin 2	8
NM_001114173	CTSC	cathepsin C	7.8
NM_001199893	ACTG2	actin, gamma 2	7.8
NM_002889	RARRES2	retinoic acid receptor responder (tazarotene induced) 2	7.7

Abbreviations: TMs, Tonsil-derived Mesenchymal stem cells; BMs, Bone marrow-derived Mesenchymal stem cells

Table III. Top 50 transcripts down-regulated in TMs compared to BMs

Transcript_ID	Gene_Symbol	Gene_Description	TM/BM.fc
NM_001004439	ITGA11	integrin, alpha 11	-62.3
NM_001135934	POSTN	periostin	-46.2
NM_006211	PENK	proenkephalin	-42.5
NM_015865	SLC14A1	solute carrier family 14 (urea transporter), member 1	-37.8
NM_001253900	MEST	mesoderm specific transcript	-22.2
NM_014391	ANKRD1	ankyrin repeat domain 1	-21.2
NM_002575	SERPINB2	serpin peptidase inhibitor, clade B (ovalbumin), member 2	-20.2
NM_001077427	LYPD1	LY6/PLAUR domain containing 1	-19.5
NM_001040058	SPP1	secreted phosphoprotein 1	-19
NM_002016	FLG	filaggrin	-18.7
NM_001177520	ALPL	alkaline phosphatase, liver/bone/kidney	-18.3
NM_138444	KCTD12	potassium channel tetramerization domain containing 12	-12.9
NM_001001557	GDF6	growth differentiation factor 6	-12.3
NM_006744	RBP4	retinol binding protein 4, plasma	-12.1
NM_152753	SCUBE3	signal peptide, CUB domain, EGF-like 3	-11.8
NM_001281767	EPHA5	EPH receptor A5	-11.6
NM_001270989	EPGN	epithelial mitogen	-10.8
NM_004415	DSP	desmoplakin	-10.5

NM_001845	COL4A1	collagen, type IV, alpha 1	-9.7
NM_001165252	KRTAP2-3	keratin associated protein 2-3	-9.5
NM_001293815	ALDH1A3	aldehyde dehydrogenase family 1 member A3	-9.3
NM_181709	FAM101A	family with sequence similarity 101, member A	-8.8
NM_005020	PDE1C	phosphodiesterase 1C	-8.6
NM_024812	BAALC	brain and acute leukemia, cytoplasmic	-8.2
NM_016932	SIX2	SIX homeobox 2	-8.1
NM_001282431	ARL4C	ADP-ribosylation factor-like 4C	-8
NM_005244	EYA2	eyes absent homolog 2	-8
NM_001031700	FAM198B	family with sequence similarity 198, member B	-8
NM_001283012	DEPTOR	DEP domain containing MTOR-interacting protein	-8
NM_015719	COL5A3	collagen, type V, alpha 3	-7.9
NM_003603	SORBS2	sorbin and SH3 domain containing 2	-7.8
NM_000104	CYP1B1	cytochrome P450, family 1, subfamily B, polypeptide 1	-7.7
NM_020474	GALNT1	UDP-N-acetyl-alpha-D-galactosamine:polypeptide N-acetylgalactosaminyltransferase 14 (GalNAc-T1)	-7.5
NM_001206850	NLGN4Y	neuroligin 4, Y-linked	-7.5
NM_006988	ADAMTS1	ADAM metallopeptidase with thrombospondin type 1 motif, 1	-7.4
NM_001163446	CPA4	carboxypeptidase A4	-7.4
NM_006762	LAPTM5	lysosomal protein transmembrane 5	-7.3
NM_001142480	NREP	neuronal regeneration related protein	-7.3

NM_002192	INHBA	inhibin, beta A	-7.1
NM_005556	KRT7	keratin 7	-7.1
NM_001136530	SERPINE2	serpin peptidase inhibitor, clade E, member 2	-7.1
NM_000557	GDF5	growth differentiation factor 5	-6.9
NM_018092	NETO2	neuropilin (NRP) and tolloid (TLL)-like 2	-6.6
NM_000435	NOTCH3	notch 3	-6.6
NM_001007538	SHISA2	shisa family member 2	-6.6
NR_040001	LINC01116	long intergenic non-protein coding RNA 1116	-6.4
NM_022166	XYLT1	xylosyltransferase I	-6.4
NM_014568	GALNT5	UDP-N-acetyl-alpha-D-galactosamine:polypeptide N-acetylgalactosaminyltransferase 14 (GalNAc-T5)	-6.4
NM_001080477	TENM3	teneurin transmembrane protein 3	-6.3
NM_001025368	VEGFA	vascular endothelial growth factor A	-6.2

Abbreviations: TMs, Tonsil-derived Mesenchymal stem cells; BMs, Bone marrow-derived Mesenchymal stem cells

Table IV. Top 50 transcripts up-regulated in TMs compared to AMs

Transcript_ID	Gene_Symbol	Gene_Description	TM/AM.fc
NM_001297773	PSG1	pregnancy-specific beta-1-glycoprotein 1	46.5
NM_018476	BEX1	brain expressed, X-linked 1	37.2
NM_000597	IGFBP2	insulin-like growth factor binding protein 2	24.4
NM_021570	BARX1	BARX homeobox 1	21.1
NM_002421	MMP1	matrix metallopeptidase 1	20.6
NM_000612	IGF2	insulin-like growth factor II	14.6
NM_000601	HGF	hepatocyte growth factor	14.6
NM_003012	SFRP1	secreted frizzled-related protein 1	11.2
NM_052889	CARD16	caspase recruitment domain family, member 16	11
NM_000730	CCKAR	cholecystokinin A receptor	10
NM_013409	FST	follistatin	9.5
NM_001199640	IL33	interleukin 33	9.5
NM_004932	CDH6	cadherin 6	9.3
NM_001291812	FGF5	fibroblast growth factor 5	8.9
NM_002729	HHEX	hematopoietically expressed homeobox	8.9
NM_006194	PAX9	paired box 9	8.8
NM_001256566	F2RL2	coagulation factor II (thrombin) receptor-like 2	8.7
NM_005192	CDKN3	cyclin-dependent kinase inhibitor 3	8.6

NM_001282382	PTTG1	pituitary tumor-transforming 1	8.4
NM_030965	ST6GALNAC5	ST6 (alpha-N-acetyl-neuraminyl-2,3-beta-galactosyl-1, 3)-<br>N-acetylgalactosaminide alpha-2,6-sialyltransferase 5	8.1
NM_005226	S1PR3	sphingosine-1-phosphate receptor 3	8.1
NM_001193451	TMTC1	transmembrane and tetratricopeptide repeat containing 1	8.1
NM_001255	CDC20	cell division cycle 20	8
NM_001164617	GPC3	glypican 3	8
NM_133459	CCBE1	collagen and calcium binding EGF domains 1	7.9
NM_014059	RGCC	regulator of cell cycle	7.8
NM_001253827	GALNT14	UDP-N-acetyl-alpha-D-galactosamine:polypeptide<br>N-acetylgalactosaminyltransferase 14 (GalNAc-T14)	7.3
NM_003258	TK1	thymidine kinase 1	7.3
NM_001199241	KYNU	kynureninase	7.3
NM_002825	PTN	pleiotrophin	7.3
NM_001177969	VIT	vitrin	7.2
NM_001199893	ACTG2	actin, gamma 2	7.1
NM_001277990	CXCL12	chemokine (C-X-C motif) ligand 12	7.1
NM_017449	EPHB2	EPH receptor B2	7
NM_005382	NEFM	neurofilament	7
NM_031246	PSG2	pregnancy specific beta-1-glycoprotein 2	7
NM_031453	FAM107B	family with sequence similarity 107, member B	6.8

NM_002546	TNFRSF11B	tumor necrosis factor receptor superfamily, member 11b	6.8
NM_006877	GMPR	guanosine monophosphate reductase	6.7
NM_005733	KIF20A	kinesin family member 20A	6.7
NM_001100620	TROAP	trophinin associated protein	6.6
NM_206827	RASL11A	RAS-like, family 11, member A	6.6
NM_001032280	TFAP2A	transcription factor AP-2 alpha	6.6
NR_001447	MT1L	metallothionein 1L	6.6
NM_198148	CPXM2	carboxypeptidase X (M14 family), member 2	6.6
NM_130386	COLEC12	collectin sub-family member 12	6.5
NM_053001	OSR2	odd-skipped related transciption factor 2	6.5
NM_001271368	PTGS1	prostaglandin-endoperoxide synthase 1	6.4
NM_001144996	ITGA7	integrin, alpha 7	6.3
NM_003248	THBS4	thrombospondin 4	6.2

Abbreviations: TMs, Tonsil-derived Mesenchymal stem cells; AMs, Adipose tissue-derived Mesenchymal stem cells

Table V. Top 50 transcripts down-regulated in TMs compared to AMs

Transcript_ID	Gene_Symbol	Gene_Description	TM/AM.fc
NM_000095	COMP	cartilage oligomeric matrix protein	-155.6
NM_001004439	ITGA11	integrin, alpha 11	-63.8
NM_001253900	MEST	mesoderm specific transcript	-37.5
NM_002016	FLG	filaggrin	-32.9
NM_199187	KRT18	keratin 18	-29.3
NM_001297709	MFAP5	microfibrillar-associated protein 5	-23
NM_080629	COL11A1	collagen, type XI, alpha 1	-22.9
NM_001283012	DEPTOR	DEP domain containing MTOR-interacting protein	-21.2
NM_001293815	ALDH1A3	aldehyde dehydrogenase family 1 member A3	-20.5
NM_032532	FNDC1	fibronectin type III domain containing 1	-19.4
NM_000362	TIMP3	TIMP metallopeptidase inhibitor 3	-19.4
NM_001244889	UNC5B	unc-5 homolog B	-19
NM_007281	SCRG1	stimulator of chondrogenesis 1	-18.8
NM_001080477	TENM3	teneurin transmembrane protein 3	-18.4
NM_001855	COL15A1	collagen, type XV, alpha 1	-15.9
NM_001135	ACAN	aggrecan	-15.6
NM_001003396	TPD52L1	tumor protein D53	-14.1
NM_004936	CDKN2B	cyclin-depend ent kinase inhibitor 2B	-14

NM_139072	DNER	delta/notch-like EGF repeat containing	-13.6
NM_001031700	FAM198B	family with sequence similarity 198, member B	-13.1
NM_004750	CRLF1	cytokine receptor-like factor 1	-13.1
NM_000526	KRT14	keratin 14	-13
NM_001135934	POSTN	periostin	-12.9
NM_002345	LUM	lumican	-12.4
NM_003391	WNT2	wingless-type MMTV integration site family member 2	-11.7
NM_001935	DPP4	dipeptidyl-peptidase 4	-11.6
NM_000638	VTN	vitronectin	-11.5
NM_001079520	DACT1	dishevelled-binding antagonist of beta-catenin 1	-11.5
NM_000576	IL1B	interleukin 1, beta	-11.4
NM_001145106	FIBCD1	fibrinogen C domain containing 1	-11.4
NM_001792	CDH2	cadherin 2	-10.9
NM_003062	SLIT3	slit homolog 3	-10.8
NM_005929	MFI2	antigen p97 (melanoma associated)	-10.6
NM_018837	SULF2	sulfatase 2	-10.4
NM_001278074	COL5A1	collagen, type V, alpha 1	-10.4
NM_002575	SERPINB2	serpin peptidase inhibitor, clade B (ovalbumin), member 2	-10.2
NM_016588	NRN1	neuritin 1	-10.2
NM_001163446	CPA4	carboxypeptidase A4	-9.9
NM_001136530	SERPINE2	serpin peptidase inhibitor, clade E, member 2	-9.9

NM_005020	PDE1C	phosphodiesterase 1C	-9.8
NM_000683	ADRA2C	adrenoceptor alpha 2C	-9.7
NM_181726	ANKRD37	ankyrin repeat domain 37	-9.5
NM_002237	KCNG1	potassium voltage-gated channel, subfamily G, member 1	-9.4
NM_004415	DSP	desmoplakin	-9.4
NM_000602	SERPINE1	serpin peptidase inhibitor, clade E), member 1	-9.2
NM_005606	LGMN	legumain	-9.2
NM_001718	BMP6	bone morphogenetic protein 6	-9.2
NM_004669	CLIC3	chloride intracellular channel 3	-9.2
NM_003014	SFRP4	secreted frizzled-related protein 4	-9.2
NM_021936	PAPPA2	pappalysin 2	-9.1

Abbreviations: TMs, Tonsil-derived Mesenchymal stem cells; AMs, Adipose tissue-derived Mesenchymal stem cells
