# Supplementary material for: RNA sequencing reveals a transcriptomic portrait of human mesenchymal stem cells from bone marrow, adipose tissue, and palatine tonsils
Source: Sci Rep. 2017 Dec 7;7:17114. doi: 10.1038/s41598-017-16788-2 (PMC5719355; doi:10.1038/s41598-017-16788-2)

RNA sequencing reveals a transcriptomic portrait of human mesenchymal stem cells from bone marrow, adipose tissue, and palatine tonsils.

Kyung-Ah Cho[1], Minhwa Park[1], Yu-Hee Kim[1], So-Youn Woo[1], and Kyung-Ha Ryu[2,*]

[1]Department of Microbiology, College of Medicine, Ewha Womans University, Seoul 07985, Republic of Korea

[2]Department of Pediatrics, College of Medicine, Ewha Womans University, Seoul 07985, Republic of Korea

*Corresponding author:

Kyung-Ha Ryu, MD, PhD

Department of Pediatrics, College of Medicine, Ewha Womans University, Seoul 07985, Republic of Korea

Tel: 82-2-2650-5500

Fax: 82-2-2653-3718

E-mail address: ykh@ewha.ac.kr

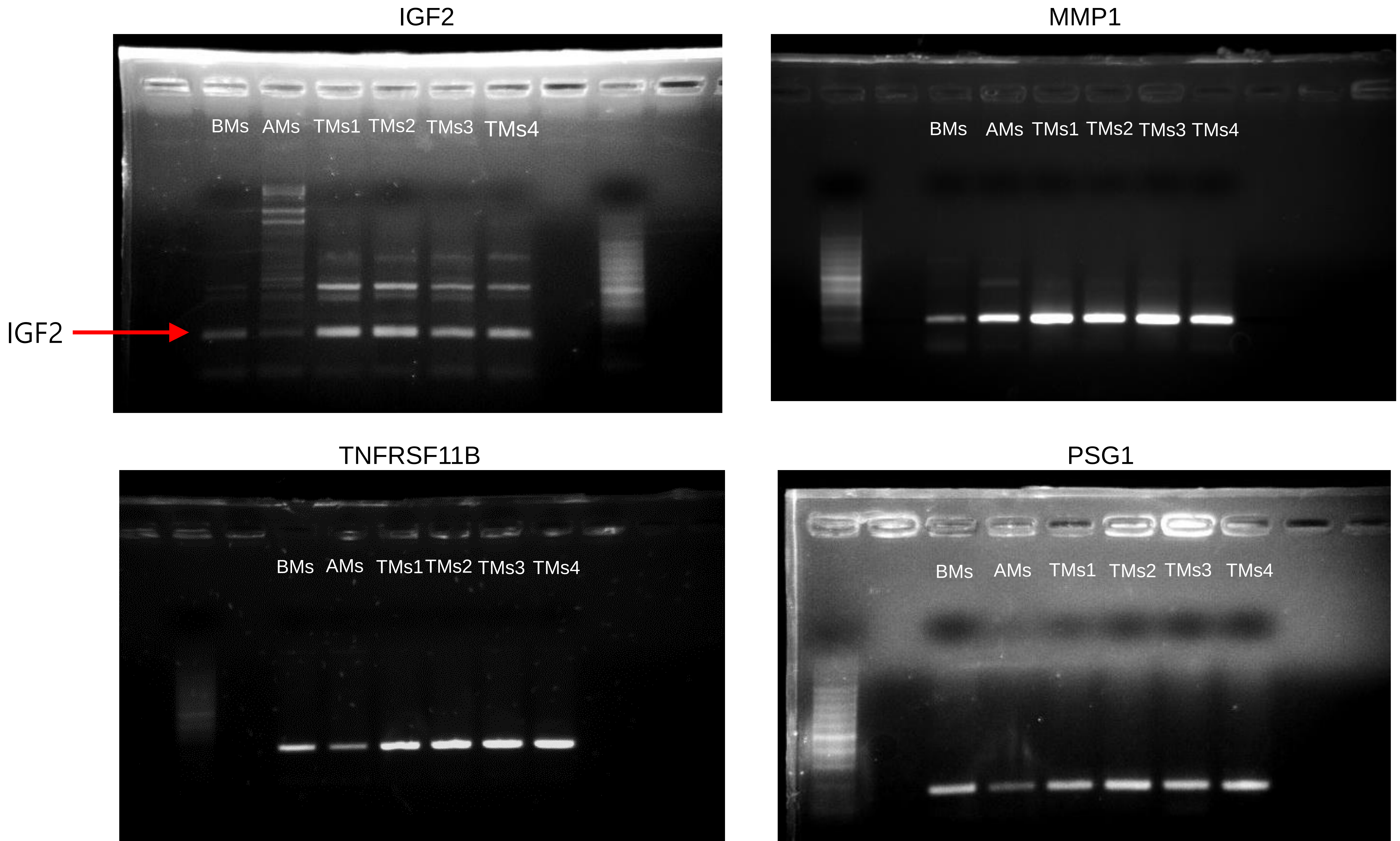
IGF2
BMs AMs TMs1 TMs2 TMs3 TMs4
IGF2
MMP1
BMs AMs TMs1 TMs2 TMs3 TMs4
TNFRSF11B
BMs AMs TMs1 TMs2 TMs3 TMs4
PSG1
BMs AMs TMs1 TMs2 TMs3 TMs4

GPC3

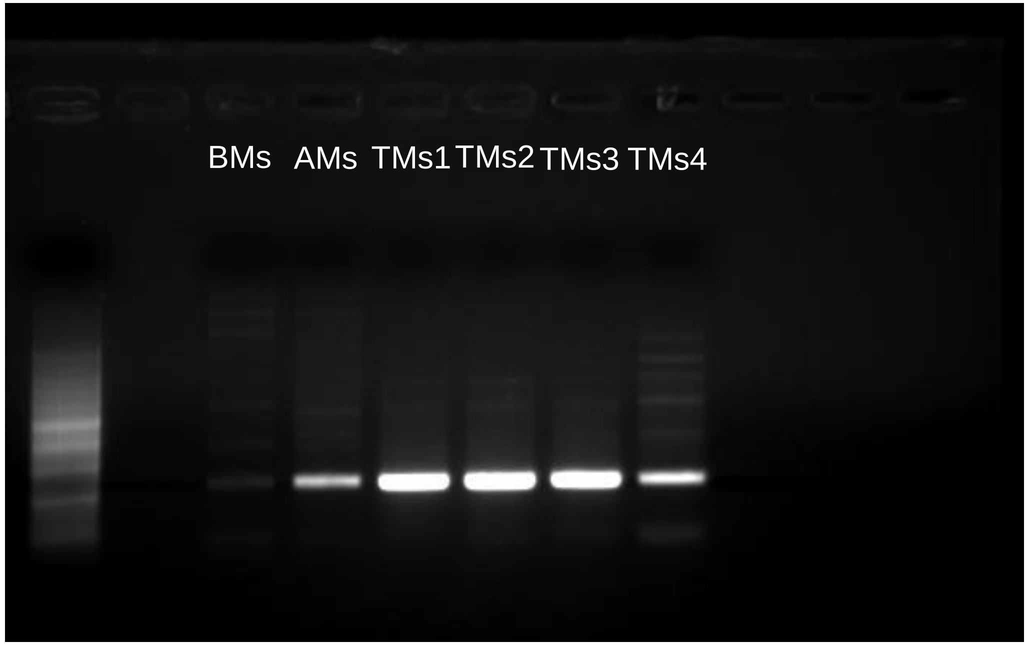

COL11A1

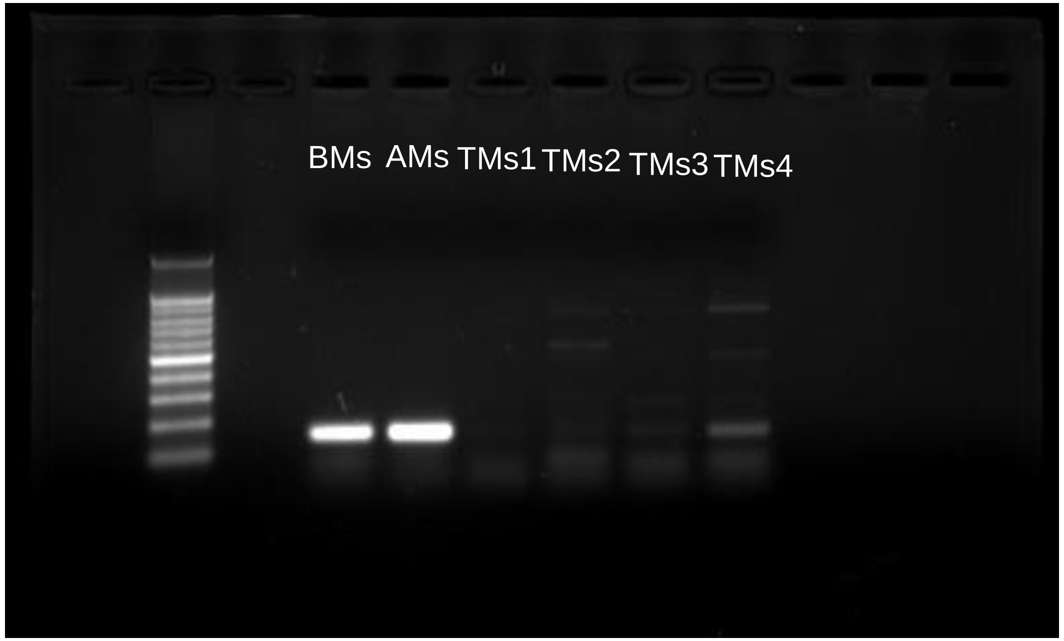

ITGA11

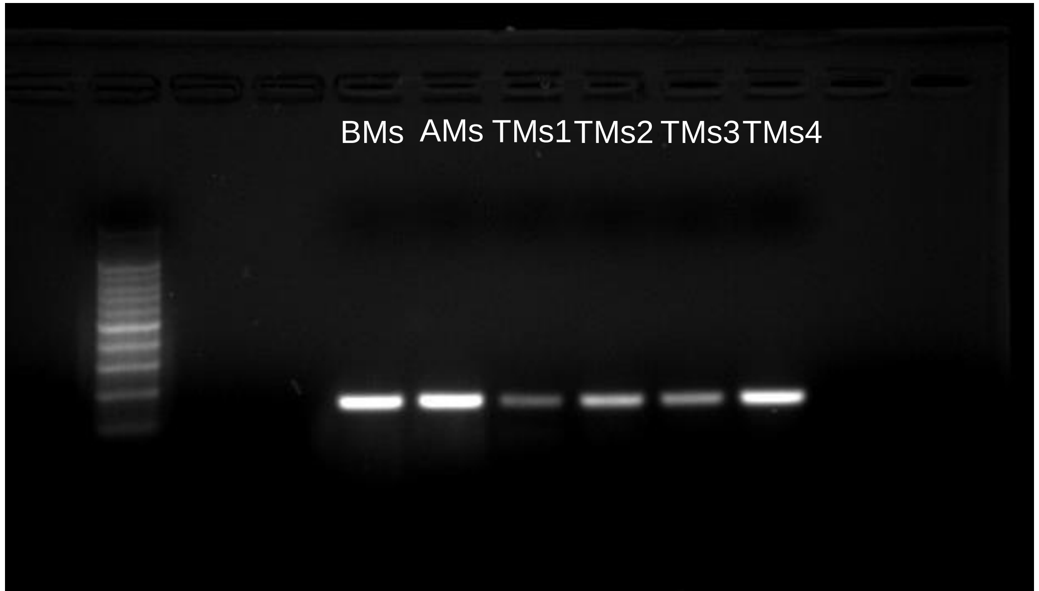

GAPDH

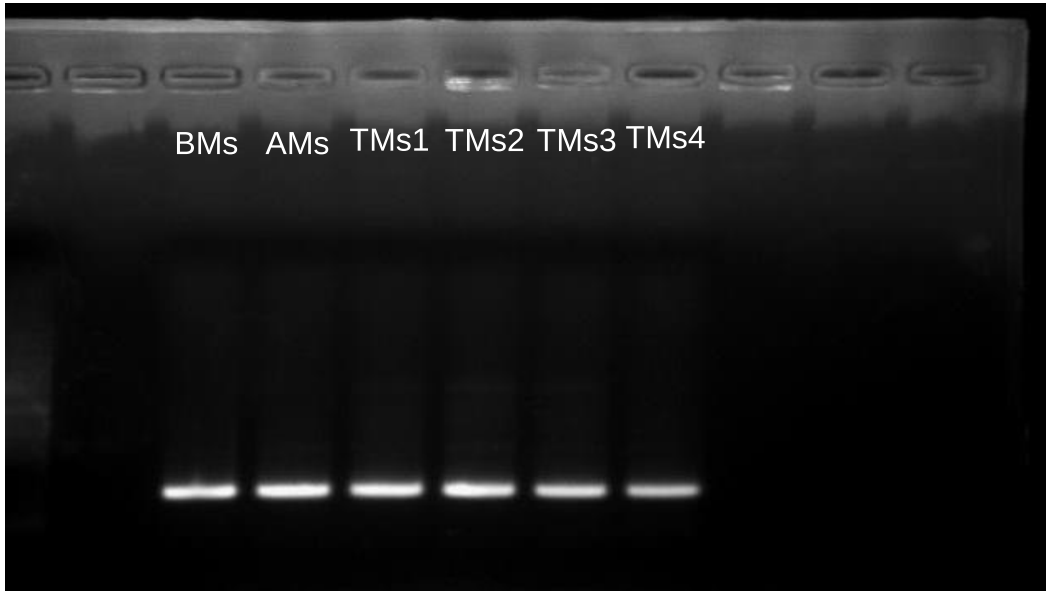

Supplement: Supplementary file 2 — RT PCR full length gel images [file 41598_2017_16788_MOESM2_ESM.pdf]
